# Structural measures of personal networks predict migrants’ cultural backgrounds: an explanation from Grid/Group theory

**DOI:** 10.1093/pnasnexus/pgac195

**Published:** 2022-09-21

**Authors:** José Luis Molina, Juan Ozaita, Ignacio Tamarit, Angel Sánchez, Christopher McCarty, H Russell Bernard

**Affiliations:** GRAFO-Department of Social and Cultural Anthropology, Universitat Autònoma de Barcelona (UAB), 08193 Bellaterra, Spain; Grupo Interdisciplinar de Sistemas Complejos, Departamento de Matemáticas, Universidad Carlos III de Madrid (UC3M), 28903 Madrid, Spain; Unidad Mixta Interdisciplinar de Comportamiento - Unidad Social (UMICSS) UC3M-UV-UZ, UC3M, 28903 Madrid, Spain; Grupo Interdisciplinar de Sistemas Complejos, Departamento de Matemáticas, Universidad Carlos III de Madrid (UC3M), 28903 Madrid, Spain; Grupo Interdisciplinar de Sistemas Complejos, Departamento de Matemáticas, Universidad Carlos III de Madrid (UC3M), 28903 Madrid, Spain; Unidad Mixta Interdisciplinar de Comportamiento - Unidad Social (UMICSS) UC3M-UV-UZ, UC3M, 28903 Madrid, Spain; Institute for Biocomputation and Physics of Complex Systems (BIFI), University of Zaragoza, 50009 Zaragoza, Spain; UC3M-Santander Big Data Institute (IBiDat), 28903 Getafe, Spain; Department of Anthropology, University of Florida (UFL), Gainesville, FL 32611, USA; School of Human Evolution and Social Change, Arizona State University (ASU), Tempe, AZ 85281, USA

**Keywords:** cultural signature, social signature, personal networks, grid/group theory, migration

## Abstract

Culture and social structure are not separated analytical domains but intertwined phenomena observable in personal networks. Drawing on a personal networks dataset of migrants in the United States and Spain, we show that the country of origin, a proxy for diverse languages and cultural institutions, and religion may be predicted by specific combinations of personal network structural measures (closeness, clustering, betweenness, average degree, etc). We obtain similar results applying three different methods (a multinomial logistic regression, a Random Forest algorithm, and an artificial neural network). This finding is explained within the framework of the Grid/Group theory that has long posed the interdependence of social structural and cultural features of human groups.

Significance Statement Drawing on a rich dataset about migrants in two countries (the United States and Spain), we predict the country of origin and, to a lesser degree, the religion of individual migrants using three different methods. This finding shows that structural and cultural dimensions are intertwined, as suggested by the Grid/Group theory. Moreover, each group of migrants exhibits a particular network pattern or “cultural signature,” much like the “social signature” that individuals uniquely exhibit in their structure of social interactions. This finding opens new avenues for studying the interdependence between social and cultural phenomena and the study of cultural diversity through a structural lens.

## Introduction

**Fig. 1.**
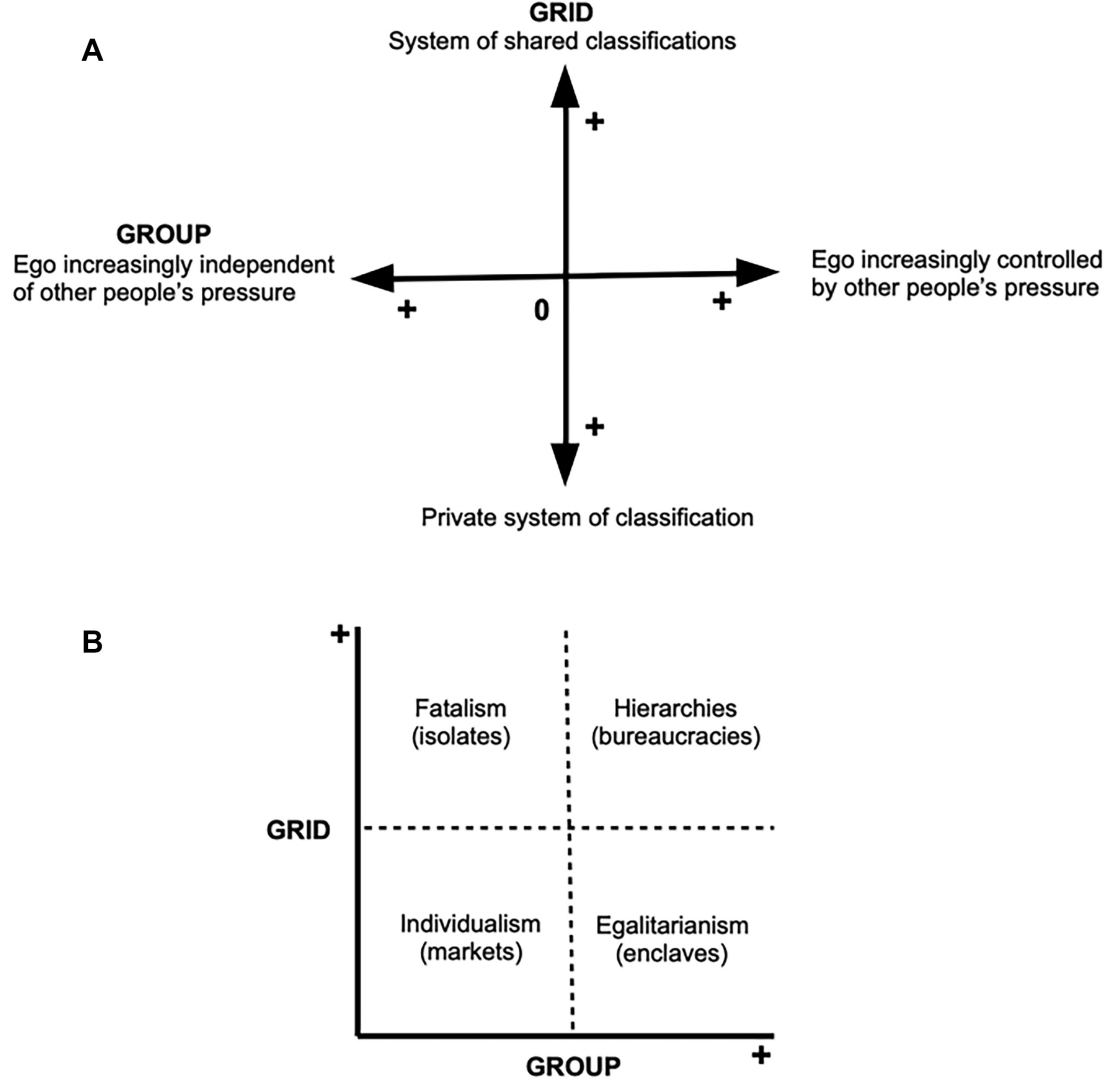
**(A)** Original formulation of the Grid/Group theory by Douglas [1996 (1973)]; and**(B)** ideal types of society (8).

The study of human societies has sought meaningful patterns of behavior either in the structure of interactions (“the social structure”) or in the ensemble of values, norms, beliefs, and material realizations of their members (“the culture”) (1, 2). Nadel (3, 4), among others (5, 6), points out the difficulty of simultaneously considering social structure and culture because status and role description are typically undertaken through formal methods, whereas the cultural content of such positions is naturally described through qualities and attributes. One of the few attempts to reconcile the analysis of both dimensions of human societies was posed by Mary Douglas and her colleagues (7–10) with Grid/Group theory (11, 12). This theory states that all human societies can be meaningfully classified along two axes: grid, or the level of control on shared classifications of the world, including the ascription of people to these categories (“what can I do”); and group, or the level of control of ego's membership in bounded groups (“who am I”; see Fig. 1A). The grid axis measures the level of control that the ensemble of values, norms, and given categories exert on the individual. A “high grid” implies that the self is bound to the ascribed social category to which it pertains. Consequently, these categories firmly constrict the range of alternative behaviors and the associated internalized values. A pariah in a caste system would be an extreme instance of this case. Conversely, a “low grid” allows individuals to choose among alternative behaviors and achieve new roles (at least in theory), typically through competition. Melanesian big men or capitalist entrepreneurs are instances of this case.

The group axis measures the level of constraint that the social unit exerts on the individual. A “high group” implies a high density of interaction among group members, long-term commitment, strong identification with the group, and defense of the corporate interests. Conversely, “low group” constraint gives “individuals the freedom to interact as individuals with other individuals,” linking loosely bounded social units. A monastic order and freelance professionals would be instances of both extreme cases, respectively. Generally speaking, both coordinates are meant to be related or “compatible”—e.g. an individualistic society (low grid/low group) will conceive nature as raw materials ready to be used, while a hierarchical society (high grid/high group; see Fig. 1B) will address access to nature in a ritualistic way through an elaborated set of prescriptions (8).

Despite the different versions of Grid/Group theory (11), and the lack of a coherent set of theoretical statements, we find that the basic assumption of the theory can be supported empirically, opening an avenue for predicting cultural variation in human societies from samples of social structures, operationalized here as ensembles of personal networks. In this vein, there are some proposals of operationalization, made by either qualitative case studies (13, 14) or by systematic cultural comparison (15). In particular, the book *Measuring Culture* (9) elaborates on a series of indicators for both group and grid dimensions for different levels of analysis (e.g. minorities, organizations, tribes, countries, and so on). For the group dimension, the authors suggest a polythetic panoply of network measures as “proximity of individuals” (closeness), “transitivity,” “frequency of interaction,” and the “boundedness of the group” (which can be measured with, among other methods, clustering, betweenness, and average degree). For the grid dimension, the authors suggest indicators intended to measure the extent of externally imposed rules, like “role specialization,” “asymmetry of role exchanges,” “role achieved or ascribed,” and the level of “accountability” in case of inadequate role performance.

The Grid/Group theory is strongly influenced by Durkheim's (16, 17) notions of the different levels of control exerted on the individuals through the system of shared classifications (which includes religion) and the formulation of four ideal types of social phenomena combining two axes: “moral regulation” (∼grid) and “social integration” (∼group). In this vein, Triandis (18) deemed the Grid/Group proposal to be one of the earlier conceptualizations of his “Individualism–Collectivism” theory, which has engendered a rich literature (19–21). Gelfand (22) reframed this proposal recently as “Tight–Loose cultural orientations,” which allow a meaningful classification of nations, states, organizations, and even personality types across the world along this axis, primarily relying on surveys. This latter proposal could be understood as similar to the grid dimension (the level of cultural control of individuals' cognitions and behavior), and also intended to enable cross-cultural comparisons. Like in this case of Gelfand we use the country of origin as a proxy of the cultural dimension. By contrast, network data available for testing the group dimension were scarce and challenging to collect until recently. Here, we use a rich set of personal network data to identify the country of origin and religion of 472 migrants from Africa and Latin America to the United States and Spain. Several authors have shown that individuals develop patterns of social interaction, or “social signatures,” that are remarkably stable through time, even with a high rate of turnover and across different channels of communication (23–25). We test the existence of such patterns at the aggregate level—cultural signatures—and interpret the results in light of the Grid/Group theory (i.e. the covariation of group and grid dimensions).

### The personal networks dataset

**Fig. 2.**
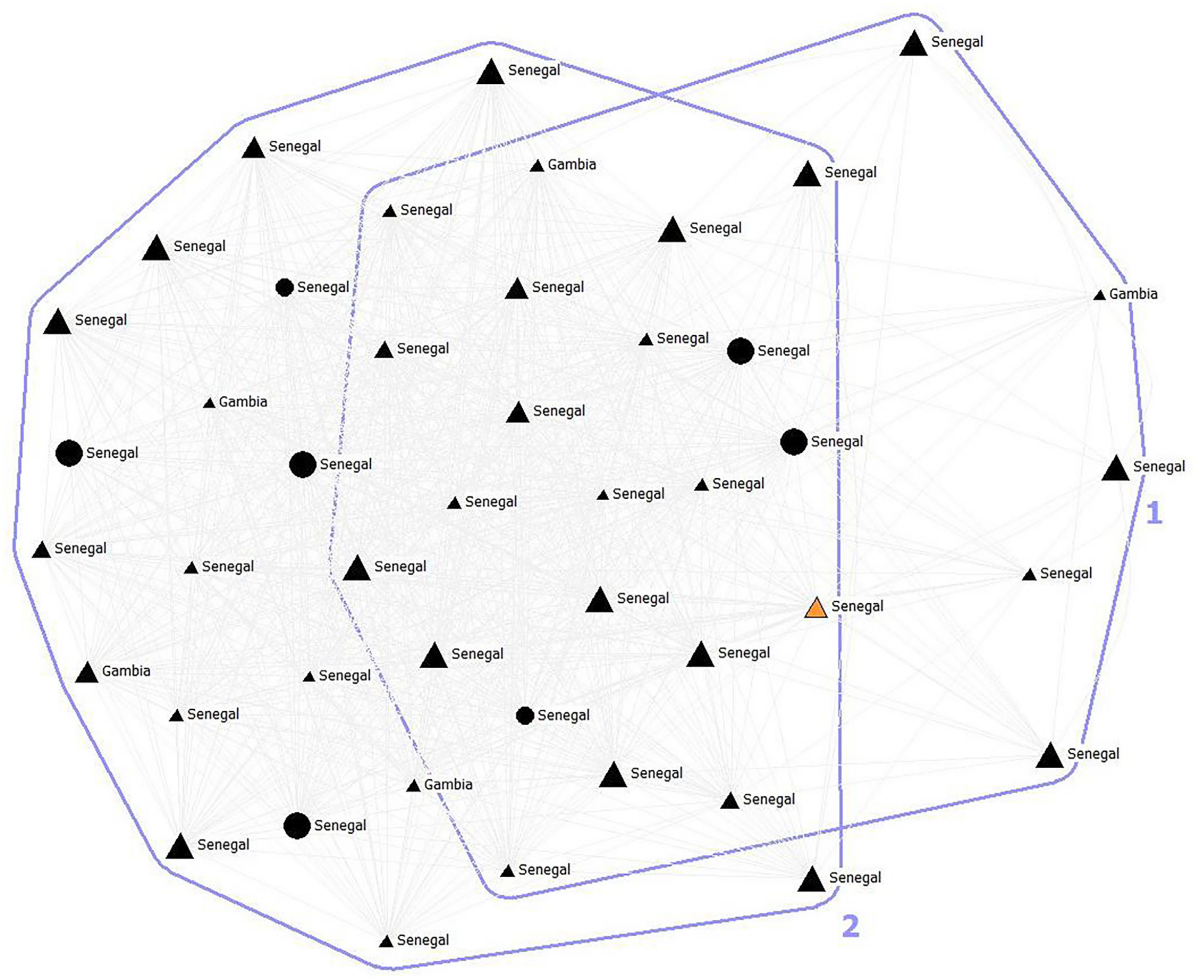
Personal network of a Senegambian migrant in Spain with 45 alters (ego is not represented in the drawing). Color shows that all the alters come from Senegambia and live in Spain (black), except one of them at the time living in Senegal (orange). Men are represented by triangles and women by circles. The size represents the frequency of interaction with ego. The program Egonet displayed in this case two clusters using the algorithm Spring Embedding.

The dataset was collected from 2004 to 2006 for the project Development of a Social Network Measure of Acculturation and its Application to Immigrant Populations in South Florida and Northeastern Spain, funded by the National Science Foundation (BCS-0417429). Personal networks were collected with the aid of the software EgoNet, in a four-module survey that lasted, on average, about 1.5 h. Questions about ego, including both demographic variables and outcome variables that we wanted to predict using 95 network characteristics. Second was a question to elicit alters or the “name generator.” For this study, we used the following prompt: “Please give us the names of 45 people you know who know you by sight or by name, with whom you have had some contact in the past two years, either face-to-face, by phone, mail or e-mail, and whom you could still contact if you had to. You can use acronyms for naming people.” This free list name generator with a fixed number of alters was designed to draw from all levels of both strong and weak ties (26–29). The third module asked egos about each alter's characteristics like gender, country of origin, period of residence in the destination country, level of education, and perceived emotional closeness between ego and alter, among other questions. Finally, the fourth module asked egos to evaluate the perceived relationship between each pair of alters with the question “How likely is it that alter X and alter Y contact each other independently of you?” The choices were “very likely,” “maybe,” and “not at all likely.” After completing the four modules, the respondents were interviewed using a visualization of their personal network as a way to ask them questions about their social context (Fig. 2) (30).

Table 1 shows the distribution of cases across country of origin. It is worth mentioning that Dominicans were interviewed in both the United States and Spain. The few cases represented by Colombians, Cubans, Guineans, Haitians, and Mexicans were aggregated under the category of “others” (*N *= 36). For the rest, the range of cases is from 66 (Moroccans) to 154 (Dominicans), with Senegambians, Argentinians, and Puerto Ricans accounting for more than 70 cases. Table 2 shows the distribution of cases by “Religion.” In this case, the category “Christian” includes Catholics (*N *= 234) and Protestants (*N *= 32); the whole sample was comprised of 49.6% Catholics.

**Table 1.** Distribution of migrant personal network cases across countries.

Origin/destination	USA		Spain		Total
	Men	Women	Men	Women	
Dominicans	47	46	37	24	154
Moroccans	0	0	25	41	66
Senegambians	0	0	54	13	67
Argentinians	0	0	38	39	77
Puerto Ricans	36	37	0	0	73
Colombians	11	3	0	0	14
Cubans	5	2	0	0	7
Guineans	0	0	6	4	10
Haitians	1	2	0	0	3
Mexicans	0	1	0	0	1
	100	91	160	121	472

**Table 2.** Distribution of migrant personal network cases across religions.

Religion	USA		Spain		Total
	Men	Women	Men	Women	
Christians	77	65	59	60	261
Muslims	2	0	71	51	124
Other	21	26	30	10	87
	100	91	160	121	472

### The analytical methods

We framed the problem as a six-class, single-label classification task. The goal of this task is to predict the country of origin of a person based on properties of his or her ego network. If these measures provide useful information to categorize a cultural trait, then the plausibility of the Grid/Group theory framework increases. As an additional result, we repeat the process with religion as the target cultural trait, including a new subset of variables.

The variables used to predict the country of origin are closeness, clustering, average degree, assortativity, and betweenness. These variables are combined with average perceived closeness with alters in origin country (closeness origin) and with alters in the destination country (note that this is a measure of the relationship ego–alter) (closeness residence). We also include the variable *µ*, that characterizes the way an individual organizes his ego network in terms of perceived closeness to the alters (31). Along with these predictors, we included the variables gender (*sex*), level of education (*educ*), and time of residence in the destination country (*fmig2*) as control variables.

For the religion variable, we apply the same set of controls and the same structural predictors. As we lacked a variable that tells us the presence of religion in the alters, we employed a new subset of variables related to the assortativity with respect to other alter attributes we have. These attributes are: the sex of the alters (*Asex*), race (*Arac*), type of relationship (*Arel*), tendency to talk about personal problems with ego (*Apro*), age (*Aol2*), contact frequency (*Afrq*), and closeness of the alters with ego (*Clos*). These variables are still structural because they measure the preferential attachment between alters with the same values for these variables, revealing the relevance of these factors in the social vicinity of ego.

The set of variables we just described serve us as predictors that we leverage using different algorithms:

*Linear Models*: We used a multinomial logistic regression (32) (MNL). This model predicts the probability of a certain class-distributed variable using the functionality of a traditional logistic regression—i.e. a sigmoid function to determine probability based on certain inputs. Its main advantage is the interpretability, as the coefficients may be directly linked to the influence of a factor in the probability of belonging to a certain class. We used this model with two different procedures: an inferential part where we used the whole dataset to fit the model and understand its parameters, and a predictive part where we try its predictive power on unseen data (i.e. the test set) and provide an accuracy metric.
  The results of the MNL were analyzed via the LLR *P*-value, the pseudo *R*-squared and the comparison between log-likelihood and LL-null. The latter will let us distinguish how good our model is by comparing it with a model without predictors. Meanwhile, the LLR *P*-value provides us, like other *P*-values, a way to compare the validity of our hypothesis. These measures are standard and their interpretation can be found in several textbooks (33). The pseudo *R*-squared tell us about the fit (34) and its value tells us about the improvement between a null and the current model, providing the same kind of interpretation R2 metric provides but within a different range of values.
*Nonlinear Models*: We tried two standard nonlinear modeling techniques, a Random Forest and a simple Neural Network (32, 35, 36). The accuracy of both methods is comparable, so we focus our description here on the one providing better results, the Random Forest. As part of the data preparation, we randomized the dataset and split it into two groups, 80% for the train data and 20% for the test data. Then the data were standardized to have 0 mean and 1 SD. Lastly, a standard hyperparameter tunning was made using k-cross fold validation with *k* = 5. 198.

To better calibrate how good the results are, we compare their performance with some dummy classifiers: a uniform dummy classifier that classifies all migrant origins with an equal probability; a stratified dummy classifier that assigns probabilities depending on its representativeness; and, finally, a “most frequent” dummy classifier that always selects the most frequent class.

To analyze the importance of the different features used by the Random Forest, we leveraged the SHAP (Shapley additive explanations) values (37). This tool was developed to compute the Shapley values, a concept derived from Game Theory to allocate surpluses in coalition games in a fair way. This solution has been reconverted to an analysis tool of global interpretability of machine learning and tree models, where surpluses are successes and the players are the features. A positive (negative) SHAP value indicates that the feature (in this case, the probability of belonging to a specific country or religion) is reinforced (diminished) by the variable. The distribution of these values shows us which variables contribute more to the model's prediction, ordered accordingly.

## Results

Let us begin introducing the inference results from the MNL. In Table 3, we show the coefficients and SE of the most important features for each country of origin. These coefficients are usually interpreted in terms of ratios, representing the relative probability of belonging to a group compared to the group of reference, which in this case is the group of people from different countries (“Others”). Therefore, it measures the importance of the variable to determine a certain country of origin (or religion).

**Table 3.** Results of the multinomial logistic regression.

MLN	Dominicans	Puerto Ricans	Argentinians	Moroccans	Senegambians
Closeness	3, 6 ± 1, 3	3, 0 ± 1, 6	3, 4 ± 1, 3	2, 6 ± 1, 3	1, 9 ± 1, 3
Betweenness	−0, 77 ± 0, 32	−0, 64 ± 0, 38	−0, 68 ± −1, 35	−1, 46 ± 0, 41	−0, 90 ± 0, 40
Clustering	−0, 64 ± 0, 25	−0, 56 ± 0, 26	1, 01 ± 0, 34	0, 26 ± 0, 29	0, 11 ± 0, 29
Average degree	−4, 0 ± 1, 35	−3, 1 ± 1, 6	−6, 3 ± 1, 4	−4, 6 ± 1, 4	−2, 9 ± 1, 4
Closeness origin	1, 10 ± 0, 32	0, 68 ± 0, 26	0, 19 ± 0, 35	1, 03 ± 0, 40	0, 07 ± 0, 41
Closeness residence	−0, 08 ± 0, 27	−0, 46 ± 0, 30	0, 50 ± 0, 34	0, 27 ± 0, 32	−0, 04 ± 0, 29

Row labels show the most relevant network measures, while column labels represent nationalities. Numbers represent coefficients and their SD .

The coefficients help us identify the network factors that differentiate each origin. There are some common characteristics to all the ego networks in our dataset, such as a high value of closeness and a low value of Betweenness. But some variables also help us distinguish different groups: betweenness ordered bottom up can make a qualitative difference between being part of an African group of migrants (Moroccan, Senegalese) and the rest of the dataset. In the same vein, closeness differentiates the Senegambian group from everyone else. Finally, the main differences can be checked out with the clustering values. This variable differentiates the group of Argentinians from the ones of the Caribbean and the ones from Africa. Table 4 shows the value of the LLR *P*-value virtually zero, which suggests that our model is much preferable to a null one.

**Table 4.** Summary results of the multinomial regression.

	Nationality	Religion
Log-likelihood	−626.76	−356.88
LL-null	−797.81	−467.51
LLR *P*-value	1.29 ×10^−49^	4.03 × 10^−22^
Pseudo *R*-squared	0.2144	0.2366

This can be also be corroborated with the value of the Pseudo *R*-squared, which interpreted together with the low *P*-value (33), allows us to state that these models capture relevant information.

We now present the results from the nonlinear models formerly defined and compare them with dummy classifiers. Table 5 presents a summary of the accuracy metrics for the Random Forest classifiers for the variables nationality and religion. The Random Forest model provides a 47% improvement over the best dummy model for country of origin—we note that a MNL model trained on the same data produces a 16% increase over the same null model. This supports the claim that these personal networks contain information about the definition of the specific migrant group.

**Table 5.** Accuracy parameters for the Random Forest model and the dummy classifiers.

Classifiers	RF	Uni	Str	MF
Nationality accuracy	0.48	0.16	0.20	0.32
Religion accuracy	0.58	0.33	0.41	0.55

The keys for the table are RF (Random Forest), Uni (uniform), Str (stratified), and MF (most frequent).

The case of Dominicans, which are present in the two destination countries (the United States and Spain), allows us to test further the Random Forest model's ability to classify by nationality from personal network measures adequately. Table 6 presents the accuracy parameter for both cases: all Dominicans, irrespective of the destination country, and Dominicans in each country, as separated classes. The results show that the prediction is better for the first case (83% for all Dominicans) than for Dominicans considered separately (37%), pointing to the fact that common network traits shared by this migrant group in two different destination contexts provide additional information to the classifier (see the separated analysis in Supplementary Material).

**Table 6.** Random forest model accuracy parameter for the case of Dominicans.

Nationality	RF
All Dominicans	0.83
Dominicans split into two classes	0.37

However, the results are less efficient for the task of predicting religion, with only a 5% over the most frequent classifier. We note that Christians are over-represented in the sample, accounting for half of the population. Despite the lesser statistical relevance achieved with this method, the classifier learns some genuine features of this cultural variable.

**Fig. 3.**
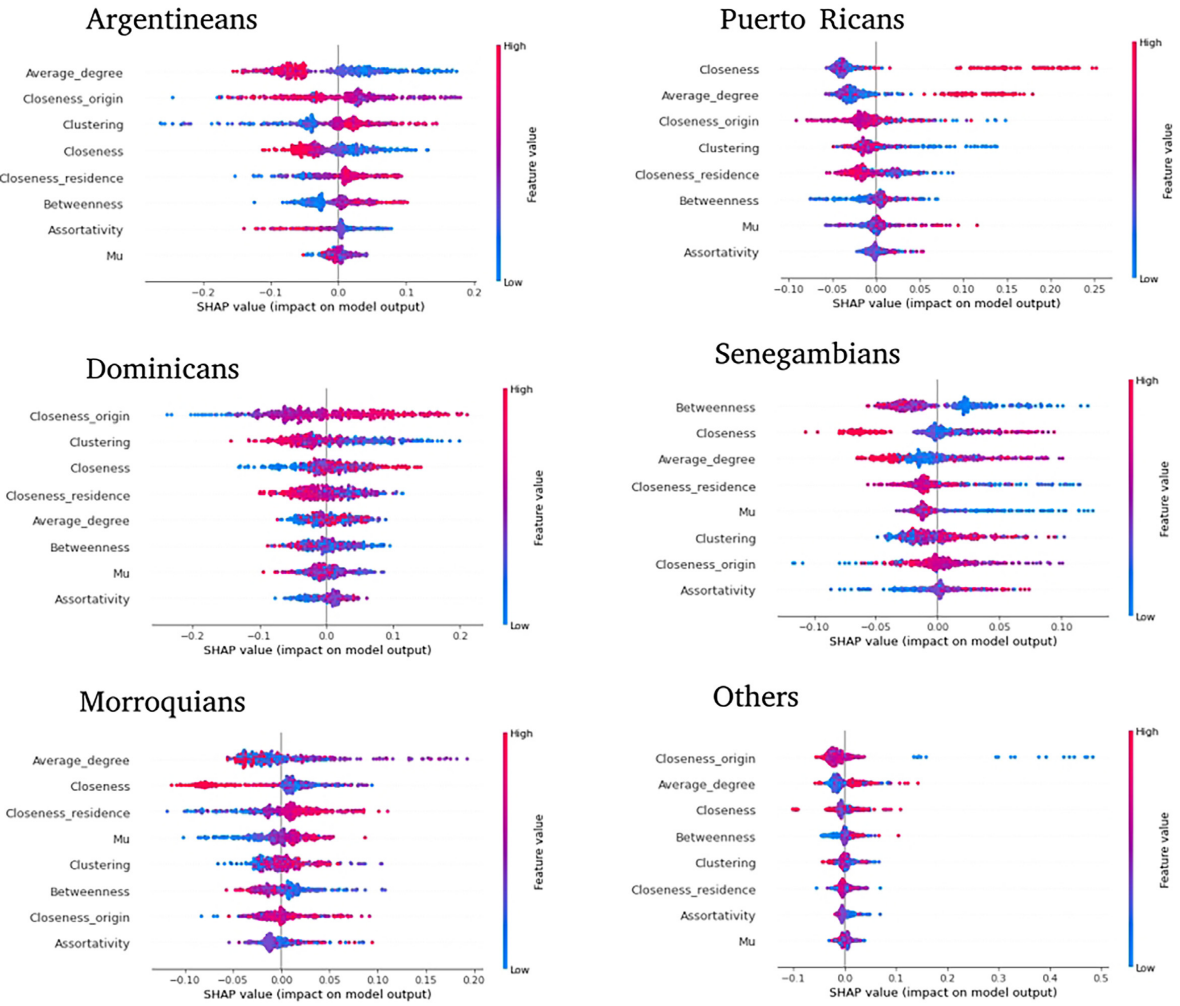
SHAP plots of the predictor variables aggregated by country. The color indicates the value of the feature, relating its value distribution with the distribution of SHAP values. The correlation between these two distributions characterizes a given nationality.

Interpreting nonlinear models can be challenging, but there are powerful techniques to overcome this issue. In Fig. 3, we show the summary plots of SHAP values distribution for distinct features and each migrant group. For each one of the plots, each *x*-axis is an individual feature, each point is an individual from our dataset whose color indicates the value of the feature. A high, positive (negative) SHAP value indicates that the feature contributes positively (negatively) to classify on a given country of origin. If the points are also separated by color, the interpretation is the existence of a relationship between the value of the feature and its contribution to the final prediction. It is important to note that the features in the plot are the most relevant ones (the ones with the highest SHAP values).

Fig. 3 presents the observed variation among nationalities. In the case of Argentinians, the main predictor is a low value of average degree combined with a high value of clustering, which reinforces each other. Puerto Ricans have a high closeness value, average degree, and a low clustering value. Dominicans also exhibit this low clustering value, but they are closer to their origin. Senegambians have a low value of betweenness, and Moroccans have a high value of closeness with the host country. Some variables diminish the probability of belonging to a certain country: this is the case of closeness in the case of Senegambians or Moroccans. It is interesting to note that some of the relationships that appear in the SHAP values are also present in the MNL results, indicating that the nonlinear effects are indeed directionally consistent the linear ones. See, for example, the effect of the distribution of closeness values in Senegalese and Moroccans. Notice in Fig. 3, in the closeness row, that the same color (red) is at the same tie (left) of the distribution. This also happens with the clustering values between Dominicans and Puerto Ricans, betweenness with Senegalese and Moroccans, and to a lesser degree, in the clustering between Dominicans and Puerto Ricans.

In the case of religion, the results are less conclusive as the sample is not well distributed across this variable. After including assortativity as a predictor, Christian religion correlates with a low sex assortativity, while Muslim religion correlates with a high sex assortativity, results aligned with the sex segregation rules of each case (see Supplementary Material).

Overall, our results may be interpreted as hypothesized at the beginning of this paper—i.e. that a specific combination of structural measures of the ensembles of alter–alter matrices predicts the country of origin of each migrant group.

## Discussion

The following findings suggest that looking at the group dimension with the aid of personal networks and structural measures can identify traces of cultural institutions (the grid dimension), which varies across countries and regions with common historical background. Obviously, there is no exact match between specific network measures and cultures, but different network profiles are associated to cultural backgrounds.

- Structural measures of personal networks predict a cultural trait like “country of origin” or “religion.” The overall results from both methods (MNL and Random Forest, Tables 3 and 5) provide support for the idea that it is possible to predict the country of origin from network measures reliably and, with less accuracy, the religion except for the case of Muslims, a category less mixed than the other two (“Christian” and “Other”). This finding can be interpreted within the Grid/Group theory framework, which states the interdependence of social structures and cultural phenomena. In this vein, personal networks can be conceived as samples of social structures, which reflect to some extent formal and informal cultural institutions (i.e. kinship and the gender/sex system, religious cults, education, political organization, and so on).
  For instance, the low average degree and the high clustering of the personal networks of Argentinians in our dataset may reflect a more individualistic society (“low group”) compared with Senegambians (see Fig. 1), which exhibit a high closeness among the alters (“high group”). We expect to find a different set of corresponding cultural institutions for each case (like kinship, religion, ethnic identification, and so on). Interestingly, this “compatibility” of both dimensions of human groups claimed by the theory limits the range of variation of social diversity.
- Each migrant group exhibits a specific network pattern. The research literature on “social signature” draws on the cognitive mechanisms that underpin the hierarchical structuring of personal networks. The Mu (μ) variable measures this “regime.” The relative low variability of Mu values across groups (from −3.83 to 2.30) supports the existence of such constraints for all cases. But, while the “social signature” literature focuses on the allocation of time by ego to interaction with alters (the ego–alter matrix), we draw in this paper on the alter–alter matrix measures for identifying specific cultural traits. Specifically, each migrant group exhibits a particular combination of structural measures. For instance, the Dominicans’ combination of low clustering and high closeness with origin may match with the importance of softball competition in the migrant community, which, at least in Spain, fostered intra-group interactions and the strong identification with origin. For the Moroccan case, the high perceived closeness with the alters living in Spain can be interpreted as a minority sharing a common Islamic culture in a European country. Finally, Puerto Ricans’ high closeness and average degree may suggest the existence of a densely knitted Latino minority in the United States.
  Last but not least, the focus on personal network data as samples of social structures highlights this meso-level analysis's potential for addressing theoretical questions about society and culture as posed by Breiger (38) and Lazega (39), among others.

## Conclusions

The analysis of structural measures of migrant personal networks shows that it is possible to infer not just their country of origin or their religion, but that each of them exhibit a particular combination of network measures compared with others. This finding is relevant theoretically because it shows an avenue of research oriented to overcome the duality of culture and structure and support the Grid/Group analytical framework.

Three different methods (MNL, Random Forest, and a neural network) account for similar results. These results suggest that personal networks can be conceived of as samples of the social structures that frame the group dimension, capturing the effects of cultural institutions (the grid dimension) in the alter–alter matrix of interactions. These results are especially relevant nowadays where social interactions are routinely registered by a myriad of digital systems (40), the group dimension.

Following the theory, we could predict cultural variation from a structural lens. We are also aware of the limitations of this approach. First, Grid/Group analysis or Cultural Theory is neither a coherent nor a developed set of clear theoretical statements and measurements. Nevertheless, we suggest that our intent to predict cultural variation from personal network structures (the “group” dimension) may complement the “Individualism–Collectivist”/“Tight–Loose” literature, which could be considered as a measure of the “grid” dimension (i.e. the level of control of social norms on individuals, as defined by Gelfand) (20). Predictions from one theory could be tested by the other and vice versa.

Second, we acknowledge the limitation of taking migrants from a given country as representatives of a culture because these migrants can be over-represented from specific minorities or groups in the sending country, which generally are not culturally homogeneous. In the same vein, the variable religion is oversampled for Christians, which hinders the learning capacity of some analytical procedures. However, we think that the country of origin is a reasonable proxy for diverse “cultural institutions” understood as the mainstream sets of values, models, and rules shared by people from a given region in all its diversity. For instance, language, kinship, and indeed religion do vary across migrant groups, and this variation should leave distinctive traces in the personal networks. Interestingly, we showed that migrant groups from wide geographical areas such as the Caribbean or West Africa shared some structural features, which can point to the imprint left by common cultural institutions.

Third, despite our controls by gender, age, and time of residence in the destination country, research on personal networks shows that individual characteristics, like socioeconomic background and the specific point in the life course, have consequences for both composition and structural measures (27–29).

Consequently, we should expect more variation on samples drawn to represent the general population of a given country than in samples from migrant groups.

Finally, structural measures are closely related, which may lead to misinterpretation of the results, even after controlling for multicollinearity.

We hope that more nuanced measures of the group/grid dimensions can help develop a better understanding of the interdependencies between social structures and cultural phenomena beyond simple determinism, which can be tested cross-culturally (41).

## Supplementary Material

pgac195_Supplemental_Files

## Data Availability

All data are included in the manuscript and/or supporting information.
