# Correction to: Structural measures of personal networks predict migrants’ cultural backgrounds: an explanation from Grid/Group theory

**DOI:** 10.1093/pnasnexus/pgad469

**Published:** 2024-01-30

This is a correction to: José Luis Molina, Juan Ozaita, Ignacio Tamarit, Angel Sánchez, Christopher McCarty, H Russell Bernard, Structural measures of personal networks predict migrants’ cultural backgrounds: an explanation from Grid/Group theory, *PNAS Nexus*, Volume 1, Issue 4, September 2022, pgac195, https://doi.org/10.1093/pnasnexus/pgac195

During a retroactive audit conducted by *PNAS Nexus*, it was discovered that this paper was missing a statement acknowledging compliance with the *PNAS Nexus* Human and Animal Participants and Clinical Trials policy:

The University of Florida IRB 02 approved the project under protocol #2004-U-480. Data obtained from participants was collected by gaining prior and written informed consent, both in Spain and the USA.

This error has been corrected in the original article.

