# Supplementary material for: Structural measures of personal networks predict migrants’ cultural backgrounds: an explanation from Grid/Group theory
Source: PNAS Nexus. 2022 Sep 21;1(4):pgac195. doi: 10.1093/pnasnexus/pgac195 (PMC9802079; doi:10.1093/pnasnexus/pgac195)
Supplement: pgac195_Supplemental_Files [file pgac195_supplemental_files.zip › PNASNEXUS-PNASNEXUS-2022-00039-T-s03.pdf]

Analysis

December 30, 2021

1 Analysis

In this notebook we will take the data from the *Ego networks* notebook and make an analysis with the three different methods: a multinomial logistic model, a random forest method an an artificial neural network. First, we will load the data, we will check for outliers and then we will prepare and format the predictors in order to apply each one of these methods. The first step is loading the libraries, in this case we will use the standard numpy, pandas, matplotlib and seaborn for manipulating and plotting the data. In order to apply the different techniques of analysis, we will use sklearn, statsmodels and tensorflow.

[1]:
```
import pandas as pd
import matplotlib.pyplot as plt
import numpy as np
import seaborn as sns
# Sklearn
from sklearn.ensemble import RandomForestClassifier
from sklearn.model_selection import train_test_split, cross_validate,␣
↪cross_val_predict
from sklearn.metrics import classification_report, confusion_matrix,␣
↪accuracy_score
from sklearn.dummy import DummyClassifier
from sklearn.preprocessing import StandardScaler
from sklearn.model_selection import GridSearchCV
# Statsmodels
import statsmodels.formula.api as smf
from statsmodels.api import MNLogit



# Just to print prettier. Uncomment to see all (not important) warnings
import warnings
warnings.filterwarnings('ignore')

%matplotlib inline
```

1.1 Load data

The next step is loading the .csv file from the previous notebook. Then we will select the columns we will use for the analysis, as the notebook contains a lot of information of the egos not related to the structural properties of their networks. Then we will map the categorical columns to a numerical encoding in the columns of : *Subject origin*, *Subject residence*, and *Regime*.

[2]:
```
### Read data
df_2 = pd.read_csv('Redes_2.csv')
### Drop Unnecessary Variables
df_2.drop('Unnamed: 0',axis=1, inplace=True)

###Take the necessary ones
df = df_2[df_2.columns[0:17]]
df['EDUC'] = df_2['EDUC'].copy()
df['FMIG2'] = df_2['FMIG2'].copy()
df['SEX'] = df_2['SEX'].copy()
df['RELG'] = df_2['RELG'].copy()

### The numerical encoding
#not_apply = ['Subject_origin','Subject_residence','Regime']
not_apply = ['Subject_origin','Subject_residence']
diccs = [0]*len(not_apply)
i = 0
for col in not_apply:
        uniques = list(df[col].unique())
        diccs[i] = {uniques[j]:uniques.index(uniques[j]) for j in␣
 ↪range(len(uniques)) }
        df[col] = df[col].map(diccs[i])
        i+=1
df.columns = df.columns.str.replace(' ', '_')
### Reset the datatype of the columns
df['Subject_origin'].astype('int64')
df['Subject_residence'].astype('int64')
#df['Regime'].astype('int64')
df.dropna(inplace=True)
```

1.2 Prepare and explore data

We make an overview of the main statistics of the data and the properties we have generated in the past notebook.

[3]:
```
df.describe(include='all')
```

[3]:
```
        Subject_num  Subject_origin  Subject_residence          Mu   Regime  \
count    473.000000      473.000000         473.000000  473.000000      473
unique          NaN             NaN                NaN         NaN        3
top             NaN             NaN                NaN         NaN  Unclear
```

```
freq            NaN             NaN                NaN         NaN      222
mean      48.919662        4.997886           0.596195   -0.743170      NaN
std       39.256840        2.781978           0.491179   13.524199      NaN
min        1.000000        0.000000           0.000000 -294.081935      NaN
25%       17.000000        2.000000           0.000000   -0.299994      NaN
50%       42.000000        5.000000           1.000000   -0.111711      NaN
75%       66.000000        8.000000           1.000000    0.100436      NaN
max      161.000000        9.000000           1.000000    2.302179      NaN

        Average_degree  Betweenness  Closeness  Load_centrality  \
count       473.000000   473.000000 473.000000       473.000000
unique             NaN          NaN        NaN              NaN
top                NaN          NaN        NaN              NaN
freq               NaN          NaN        NaN              NaN
mean         23.910303     0.015374   0.687343         0.014964
std          13.574373     0.011329   0.216891         0.011674
min           2.628571     0.000023   0.130665         0.000023
25%          12.818182     0.006953   0.532497         0.005732
50%          19.066667     0.014024   0.632030         0.013907
75%          40.666667     0.020487   0.938077         0.020487
max          43.955556     0.078431   0.999012         0.078431

        Assortativity  …  Number_components  Size_largest_component  \
count      473.000000  …         473.000000              473.000000
unique            NaN  …                NaN                     NaN
top               NaN  …                NaN                     NaN
freq              NaN  …                NaN                     NaN
mean        -0.028397  …           1.162791                0.985243
std          0.213720  …           0.496737                0.053987
min         -0.695654  …           1.000000                0.500000
25%         -0.139165  …           1.000000                1.000000
50%         -0.045455  …           1.000000                1.000000
75%          0.024567  …           1.000000                1.000000
max          0.974478  …           6.000000                1.000000

        Closeness_residence  Number_residence  Closeness_origin  \
count            473.000000        473.000000        473.000000
unique                  NaN               NaN               NaN
top                     NaN               NaN               NaN
freq                    NaN               NaN               NaN
mean               2.766545         12.562368          2.495494
std                1.232411         10.960871          0.876297
min                0.000000          0.000000          0.000000
25%                2.263158          4.000000          2.062500
50%                3.000000         10.000000          2.560976
75%                3.600000         19.000000          3.052632
max                5.000000         66.000000          4.883721
```

```
        Number_origin        EDUC        FMIG2         SEX        RELG
count      473.000000  473.000000   473.000000  473.000000  473.000000
unique            NaN         NaN          NaN         NaN         NaN
top               NaN         NaN          NaN         NaN         NaN
freq              NaN         NaN          NaN         NaN         NaN
mean        26.670190    3.509514    83.097252    1.448203    1.000000
std         13.596995    1.432231   395.983269    0.497836   11.438857
min          0.000000    1.000000     0.000000    1.000000  -99.000000
25%         17.000000    2.000000     0.000000    1.000000    1.000000
50%         29.000000    4.000000     0.000000    1.000000    2.000000
75%         38.000000    4.000000     0.000000    2.000000    2.000000
max         72.000000    7.000000  2018.000000    2.000000    6.000000

[11 rows x 21 columns]
```

Some values of `mu` are way out of range (min = -294). This is clearly from divergences in the model. We mark observations greater than 10 (in absolute value) as `nan` and then drop `nan`.

[4]:
```
# Clean estimates for mu
df['Mu'] = df['Mu'].apply(lambda x: np.nan if x < -100 else x)
df['Mu'] = df['Mu'].apply(lambda x: np.nan if x > 100 else x)
df.dropna(inplace = True)
```

1.3 Group some nationalities in others group

We keep only classes with more than 50 observations. The rest of the classes will be considered as one called "others"

[5]:
```
# There are few data on several Origins
count_origins = pd.get_dummies(df['Subject_origin']).sum()
t = 50 # threshold
df['Subject_origin'] = df['Subject_origin'].apply(lambda x: 10 if (count_origins[x] < t) else x)
#pd.get_dummies(df['Subject_origin']).sum()
```

[6]:
```
### This is just to translate the encoding to the first five integers
dicc_traslation = {10:0,2:1,5:2,6:3,8:4,9:5}
dicc_final = {0:"Other",1:"Dominican",2:"PuertoRican",3:"Argentinean",4:"Moroccan",5:"Senegambian"}
df['Subject_origin'] = df['Subject_origin'].map(dicc_traslation)
```

1.3.1 Define predictors for all the inference and prediction methods

[7]:
```
predictors =␣
↪['Closeness','Clustering','Average_degree','Assortativity','Betweenness',
        ␣
↪'Closeness_origin','Closeness_residence','Number_origin','Number_residence','Mu']
target = "Subject_origin"
```

1.3.2 Define train and test split for the dataset

[8]:
```
X = df[predictors]       # independent variables
y = df[target]

test_size = 0.20 #maybe more is needed (20% is standard though)
X_train, X_test, y_train, y_test = train_test_split(X, y, test_size =␣
↪test_size, random_state = 0)


# Standard Scaler
sc = StandardScaler()
X_train = sc.fit_transform(X_train)
X_test = sc.transform (X_test)

#Define dataframe as merge of X and y
df_str = df[target].to_frame().merge(pd.DataFrame(sc.
↪fit_transform(X),columns=predictors,index=df.index), left_index=True,␣
↪right_index=True)
```

2 INFERENCE

At this point, we begin to include tools of inference, beginning by the multinomial logistic regression (MLN). The library used for this analysis is mainly *statsmodels* and the main function can be checked in this link: https://stats.idre.ucla.edu/stata/dae/multinomiallogistic-regression/

In this part of the notebook we will prepare the variables, execute the regression and save the results.

2.0.1 Fit Multinomial Logistic Model

https://www.statsmodels.org/stable/generated/statsmodels.discrete.discrete_model.MNLogit.html

[9]:
```
### Uses the list 'predictors' as independent variables
formula_predictors = ' + '.join(predictors)
target_str = target +" ~ {}"
model = MNLogit.from_formula(target_str.format(formula_predictors), df_str)
results = model.fit(maxiter=200)
```

```
Optimization terminated successfully.
         Current function value: 1.241064
         Iterations 8
```

Results

[10]:
```python
print(results.summary())
```

```
                          MNLogit Regression Results
==============================================================================
Dep. Variable:         Subject_origin   No. Observations:                  472
Model:                        MNLogit   Df Residuals:                      417
Method:                           MLE   Df Model:                           50
Date:                Thu, 30 Dec 2021   Pseudo R-squ.:                  0.2678
Time:                        04:14:04   Log-Likelihood:                -585.78
converged:                       True   LL-Null:                       -800.06
Covariance Type:            nonrobust   LLR p-value:                 1.390e-61
================================================================================
=======
   Subject_origin=1       coef    std err          z      P>|z|      [0.025
0.975]
--------------------------------------------------------------------------------
-------
Intercept              1.7954      0.267      6.729      0.000       1.272
2.318
Closeness              3.6306      1.281      2.834      0.005       1.119
6.142
Clustering            -0.6369      0.247     -2.577      0.010      -1.121
-0.152
Average_degree        -3.9920      1.321     -3.022      0.003      -6.581
-1.403
Assortativity          0.9273      0.318      2.913      0.004       0.303
1.551
Betweenness           -0.7696      0.316     -2.438      0.015      -1.388
-0.151
Closeness_origin       1.0968      0.320      3.423      0.001       0.469
1.725
Closeness_residence   -0.0885      0.272     -0.325      0.745      -0.622
0.445
Number_origin          1.4225      0.389      3.661      0.000       0.661
2.184
Number_residence       1.1709      0.349      3.360      0.001       0.488
1.854
Mu                    -0.4822      0.364     -1.325      0.185      -1.196
0.231
--------------------------------------------------------------------------------
-------
   Subject_origin=2       coef    std err          z      P>|z|      [0.025
```

```
0.975]
------------------------------------------------------------------------------------------
-------
Intercept              0.5549      0.317      1.749      0.080      -0.067
1.177
Closeness              2.9841      1.597      1.869      0.062      -0.146
6.114
Clustering            -0.5590      0.262     -2.137      0.033      -1.072
-0.046
Average_degree        -3.1214      1.602     -1.948      0.051      -6.262
0.019
Assortativity          0.8012      0.367      2.183      0.029       0.082
1.520
Betweenness           -0.6439      0.377     -1.709      0.087      -1.382
0.094
Closeness_origin       0.6880      0.263      2.613      0.009       0.172
1.204
Closeness_residence   -0.4695      0.297     -1.583      0.113      -1.051
0.112
Number_origin         -0.3230      0.353     -0.915      0.360      -1.015
0.369
Number_residence       0.6385      0.301      2.120      0.034       0.048
1.229
Mu                     0.1038      0.364      0.286      0.775      -0.609
0.816
------------------------------------------------------------------------------------------
-------
   Subject_origin=3      coef    std err          z      P>|z|      [0.025
0.975]
------------------------------------------------------------------------------------------
-------
Intercept             -0.3076      0.454     -0.677      0.498      -1.198
0.583
Closeness              3.4373      1.255      2.740      0.006       0.978
5.896
Clustering             1.0140      0.336      3.017      0.003       0.355
1.673
Average_degree        -6.2753      1.398     -4.490      0.000      -9.015
-3.536
Assortativity          0.5008      0.327      1.533      0.125      -0.139
1.141
Betweenness           -0.6794      0.354     -1.919      0.055      -1.373
0.015
Closeness_origin       0.1944      0.350      0.556      0.578      -0.491
0.879
Closeness_residence    0.5046      0.344      1.466      0.143      -0.170
1.179
Number_origin          1.1107      0.478      2.323      0.020       0.174
```

```
2.048
Number_residence        1.5030      0.420      3.580      0.000       0.680
2.326
Mu                     -0.4511      0.395     -1.143      0.253      -1.225
0.323
================================================================================
=======
   Subject_origin=4       coef    std err          z      P>|z|      [0.025
0.975]
--------------------------------------------------------------------------------
-------
Intercept               0.6399      0.319      2.008      0.045       0.015
1.264
Closeness               2.6477      1.318      2.009      0.045       0.065
5.231
Clustering              0.2606      0.292      0.893      0.372      -0.311
0.832
Average_degree         -4.5988      1.392     -3.304      0.001      -7.327
-1.871
Assortativity           0.8147      0.333      2.450      0.014       0.163
1.467
Betweenness            -1.4619      0.408     -3.579      0.000      -2.262
-0.661
Closeness_origin        1.0290      0.404      2.549      0.011       0.238
1.820
Closeness_residence     0.2744      0.318      0.862      0.389      -0.350
0.898
Number_origin           1.6100      0.479      3.359      0.001       0.671
2.550
Number_residence        2.0112      0.413      4.866      0.000       1.201
2.821
Mu                     -0.3886      0.425     -0.914      0.361      -1.222
0.444
================================================================================
=======
   Subject_origin=5       coef    std err          z      P>|z|      [0.025
0.975]
--------------------------------------------------------------------------------
-------
Intercept               0.6614      0.322      2.051      0.040       0.029
1.293
Closeness               1.8638      1.339      1.391      0.164      -0.761
4.489
Clustering              0.1122      0.293      0.383      0.702      -0.463
0.687
Average_degree         -2.8954      1.392     -2.080      0.038      -5.624
-0.167
Assortativity           0.4932      0.332      1.487      0.137      -0.157
```

```
1.143
Betweenness            -0.8954      0.405     -2.209      0.027     -1.690
-0.101
Closeness_origin        0.0702      0.411      0.171      0.864     -0.736
0.876
Closeness_residence    -0.0449      0.287     -0.156      0.876     -0.608
0.518
Number_origin           1.6061      0.487      3.296      0.001      0.651
2.561
Number_residence        0.6669      0.465      1.435      0.151     -0.244
1.578
Mu                     -0.1316      0.411     -0.320      0.749     -0.937
0.674
================================================================================
=======
```

[11]:
```python
print('pseudo r-squared = {}'.format(np.round(results.prsquared,2)))
```

```
pseudo r-squared = 0.27
```

[12]:
```python
results.llr_pvalue
```

[12]: 1.390334147058946e-61

3 PREDICTION

We train and fit a powerful non-linear (and non-parametric) machine learnin classifier to the data; a Random Forest. There are many other alternatives, but tree based metods are very powerfull and there are new techniques to help identify relevant predictors.

In this section, we want to test wether this model can outperform significantly other null (dummy) classifiers. If that is the case (which it is), it confirms the hypothesis that the predictors have relevant information about the nationalities of the subjects.

3.0.1 Train and test with MNL regression

[13]:
```python
formula_predictors = ' + '.join(predictors)
model = MNLogit.from_formula(target_str.format(formula_predictors), df_str.
 ↪loc[y_train.index])
results_prediction = model.fit(maxiter=200)
ypred = results_prediction.predict(df_str.loc[y_test.index])
y_pred =list(map(np.argmax,np.array(ypred)))
##Meter función accuracy
```

```
Optimization terminated successfully.
         Current function value: 1.196666
         Iterations 8
```

[14]:
```
from sklearn.metrics import accuracy_score
print(accuracy_score(y_test, y_pred))
```

```
0.37894736842105264
```

3.0.2 Train and tune the model using k-cross fold validation

[15]:
```
scoring = 'accuracy' #'f1_macro' # This chooses the metric to optimise during␣
↪training (there are others!)
njobs=-1                         # This the number of cores used in your cpu␣
↪(-1 means "all of them")
cv=5                             # the k in k-cross-fold validation
# RANDOM FOREST
print('\nFitting Random Forest\n')

rfc=RandomForestClassifier(random_state=0)
# Parameter combinations to explore
param_grid = {
    'n_estimators': [75, 100,300,1000],
    'max_features': ['auto', None],
    'min_samples_split' :[2,6, 10, 14],
    'max_depth' : [10, 15, 30, 50,None],
    'max_samples' : [0.5 ,0.7, None],}


CV_rfc = GridSearchCV(estimator=rfc,
                  param_grid=param_grid,
                  scoring = scoring,
                  verbose=0,
                  n_jobs=njobs,
                  cv= cv)
CV_rfc.fit(X_train, y_train)

print('\nRandom Forest:')
print('Best Score: ', CV_rfc.best_score_)
print('Best Params: ', CV_rfc.best_params_)
```

```
Fitting Random Forest


Random Forest:
Best Score:  0.5121052631578947
Best Params:  {'max_depth': 10, 'max_features': 'auto', 'max_samples': None,
'min_samples_split': 14, 'n_estimators': 300}
```

3.0.3 Evaluating the algorithm performance in the test set (unseen data)

[16]:
```
y_pred = CV_rfc.predict(X_test)
print('Confusion Matrix:\n ', confusion_matrix(y_test,y_pred),'\n')
print(classification_report(y_test,y_pred),'\n')
print('Accuracy: {0:.2f}'.format(accuracy_score(y_test, y_pred),2))
dicc_final = {0:"Other",1:"Dominican",2:"PuertoRican",3:"Argentinean",4:
 ↪"Moroccan",5:"Senegambian"}
```

```
Confusion Matrix:
  [[ 1  2  0  2  1  0]
 [ 0 19  2  4  0  0]
 [ 0  7  6  2  1  0]
 [ 0  4  0 11  1  0]
 [ 0  4  0  5  7  1]
 [ 0  6  0  4  3  2]]

              precision    recall  f1-score   support

           0       1.00      0.17      0.29         6
           1       0.45      0.76      0.57        25
           2       0.75      0.38      0.50        16
           3       0.39      0.69      0.50        16
           4       0.54      0.41      0.47        17
           5       0.67      0.13      0.22        15

    accuracy                           0.48        95
   macro avg       0.63      0.42      0.42        95
weighted avg       0.58      0.48      0.45        95


Accuracy: 0.48
```

3.0.4 Compare this performance with null models

[17]:
```
#  relative prevalence of each class
rel_prev = (y.value_counts() / len(y))
print(rel_prev)
```

```
1    0.326271
3    0.163136
2    0.154661
5    0.141949
4    0.139831
0    0.074153
Name: Subject_origin, dtype: float64
```

[18]:
```
# Uniform Dummy Classifier (classifies randomly with p = 1/6)

# If the classifier randomly guesses:
print('Acurracy of uniform dummy classifier: ',(((1/6) * y.value_counts()) /␣
↪len(y)).sum()) # = 1/6
```

```
Acurracy of uniform dummy classifier:  0.16666666666666663
```

[19]:
```
# Stratified Dummy Classifier (classifies randomly with p ~ prevalence of each␣
↪class)
print('Acurracy of stratified dummy classifier: ',(rel_prev * y.value_counts()).
↪sum() / len(y))
```

```
Acurracy of stratified dummy classifier:  0.20218687158862397
```

[20]:
```
# Most frequent Dummy Classifier (classifies always in the most frequent class)
print('Acurracy of Most freq dummy classifier: ',rel_prev.max() )
```

```
Acurracy of Most freq dummy classifier:  0.3262711864406778
```

[21]:
```
# SKLEARN versions of the dummy classifiers (to double check and for␣
↪convinience methods)

dummy = "stratified"# most_frequent, stratified, uniform
dummy_clf = DummyClassifier(strategy=dummy,random_state=0)



# Actual accuracy of the dummy in the same train-test split as the RF model
dummy_clf.fit(X_train, y_train)
dummy_score = dummy_clf.score(X_test, y_test)
print('Mean accuracy of null ' + dummy +' model: {0:.2f}'.
↪format(dummy_score),'\n')
print('Mean accuracy (in test) of RF model: {0:.2f}'.format(CV_rfc.
↪score(X_test, y_test)),'\n')
```

```
Mean accuracy of null stratified model: 0.23

Mean accuracy (in test) of RF model: 0.48
```

[22]:
```
# Confusion matrix and report of the selected dummy classifier

y_pred_dummy = dummy_clf.predict(X_test)
print('Confusion Matrix:\n\n ',confusion_matrix(y_test,y_pred_dummy),'\n')
print(classification_report(y_test,y_pred_dummy),'\n')
print('Accuracy: {0:.2f}'.format(accuracy_score(y_test, y_pred_dummy),2))
```

```
Confusion Matrix:

 [[ 1  3  1  0  0  1]
 [ 1 10  5  3  4  2]
 [ 1  7  1  1  4  2]
 [ 1  5  1  4  1  4]
 [ 2  7  2  1  3  2]
 [ 0  8  0  3  1  3]]
              precision    recall  f1-score   support

           0       0.17      0.17      0.17         6
           1       0.25      0.40      0.31        25
           2       0.10      0.06      0.08        16
           3       0.33      0.25      0.29        16
           4       0.23      0.18      0.20        17
           5       0.21      0.20      0.21        15

    accuracy                           0.23        95
   macro avg       0.22      0.21      0.21        95
weighted avg       0.22      0.23      0.22        95


Accuracy: 0.23
```

[23]:

```
# Just for reference, the results of the RF Model

y_pred = CV_rfc.predict(X_test)
print('Confusion Matrix:\n\n ', confusion_matrix(y_test,y_pred),'\n')
print(classification_report(y_test,y_pred),'\n')
print('Accuracy: {0:.2f}'.format(accuracy_score(y_test, y_pred),2))
```

```
Confusion Matrix:

 [[ 1  2  0  2  1  0]
 [ 0 19  2  4  0  0]
 [ 0  7  6  2  1  0]
 [ 0  4  0 11  1  0]
 [ 0  4  0  5  7  1]
 [ 0  6  0  4  3  2]]
              precision    recall  f1-score   support

           0       1.00      0.17      0.29         6
           1       0.45      0.76      0.57        25
           2       0.75      0.38      0.50        16
           3       0.39      0.69      0.50        16
           4       0.54      0.41      0.47        17
```

```
             5       0.67      0.13      0.22        15

      accuracy                           0.48        95
     macro avg       0.63      0.42      0.42        95
  weighted avg       0.58      0.48      0.45        95


Accuracy: 0.48
```

[24]:
```
dummy_report = pd.DataFrame(classification_report(y_test,dummy_clf.
 ↪predict(X_test), output_dict= True))

rfc_report = pd.DataFrame(classification_report(y_test,CV_rfc.predict(X_test),␣
 ↪output_dict= True))
```

Increase in prediction power (percentage with respect to null model) i.e. 100% means twice as good

[25]:
```
final_table = ((rfc_report - dummy_report)*100 / dummy_report).drop('support').
 ↪round(decimals=2)
final_table
```

[25]:
```
                0      1      2       3       4       5  accuracy  macro avg  \
precision  500.00  80.95  650.0   17.86  133.33  211.11    109.09     193.45
recall       0.00  90.00  500.0  175.00  133.33  -33.33    109.09     101.83
f1-score    71.43  84.33  550.0   75.00  133.33    7.41    109.09     104.34

           weighted avg
precision        156.79
recall           109.09
f1-score         105.54
```

This significant increases further support the claim that the predictors (based on ego-network properties) have useful information to predict the countries of origin of the individuals)

3.1 Shap Values

Shap values are a tool to interpret our random forest model, in this case. They tell us some intuition about which part of the prediction belongs to each feature.

A positive (negative) SHAP value indicates that the value (in this case, probability of belonging to a certain country) is reinforced (diminished) by the feature.

We will use 2 kind of plots at this moment. The first one one is a summary plot, a violin plot of the distribution of SHAP values. The colour indicates the value of the feature indicated at the left. This plot let us see the which features contribute the most (this is, they have high SHAP values). Features are ordered according to their contribution to the global prediction.

The second kind of plot you will see several times after the summary plot is the dependence plot.

They show the distribution of the SHAP values of a variable. The colormap plots another variable, the one the algorithm thinks it has more interaction with the current variable. It lets us distinguish between different regimes of the coloured variable.

[26]:
```
# explain the model's predictions using SHAP
##Shap values
import  shap

shap.initjs()
model = CV_rfc.best_estimator_
explainer = shap.TreeExplainer(model,X_train,check_additivity=False)
shap_values = explainer.shap_values(X_train,check_additivity=False)
```

```
<IPython.core.display.HTML object>

 98%|===================| 2216/2262 [00:40<00:00]
```

3.2 Example of summary plot

We extract the summary plots that summarizes the correlations for each nationality.

SHAP values for the dominican

[27]:
```
shap.summary_plot(shap_values[1],X_train,feature_names = predictors)
```

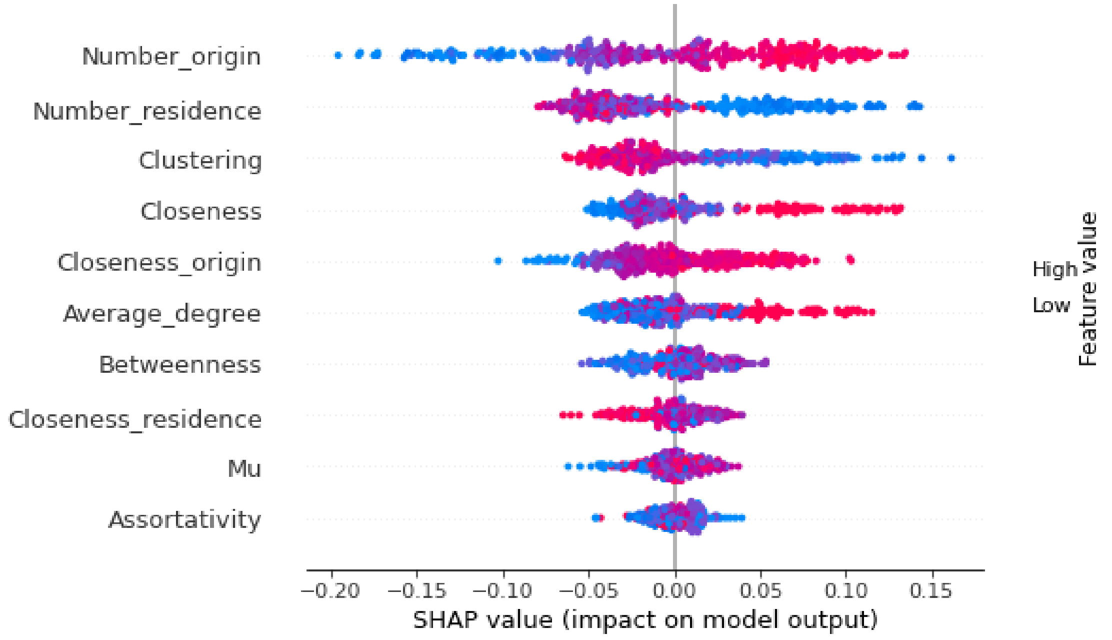

SHAP values for the Puerto Rican

[28]:
```
shap.summary_plot(shap_values[2],X_train,feature_names = predictors)
```

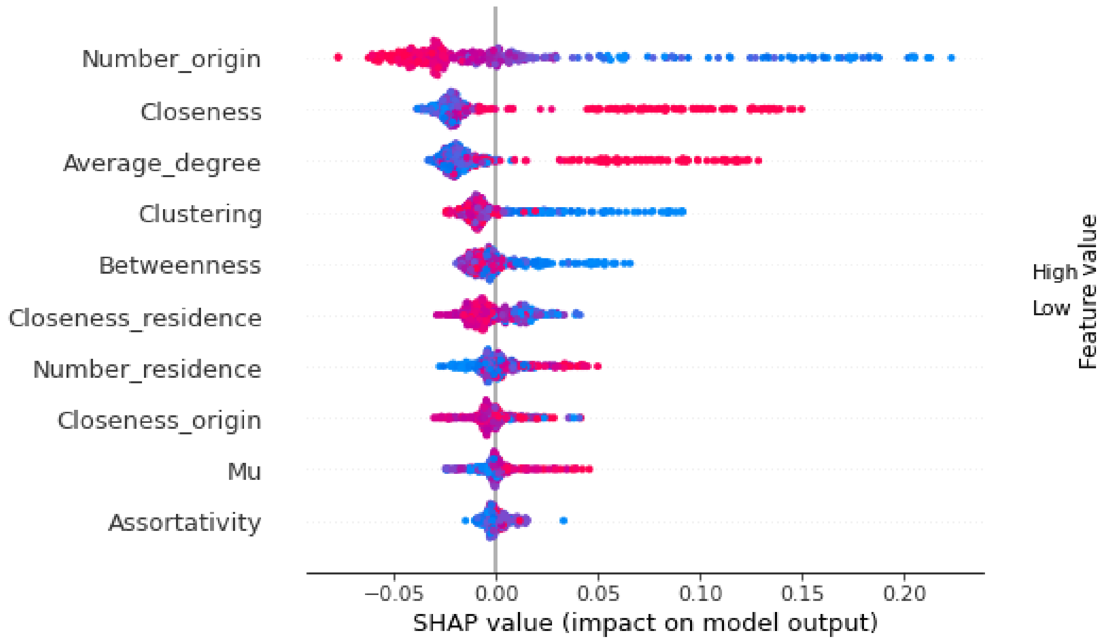

SHAP values for the argentinean

```
[29]: shap.summary_plot(shap_values[3],X_train,feature_names = predictors)
```

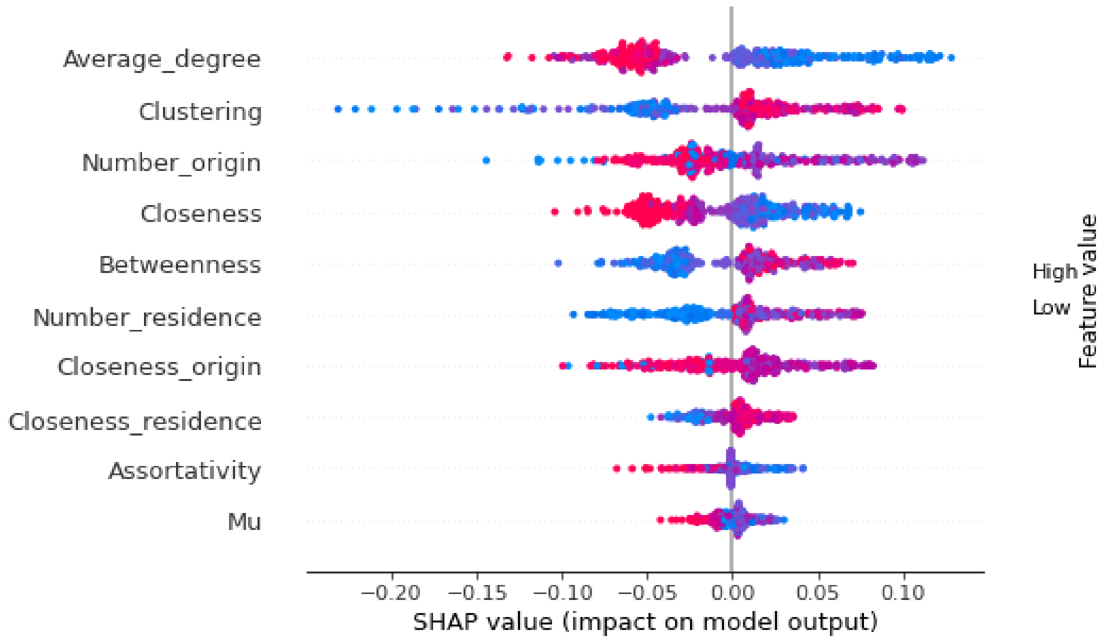

SHAP values for the moroccan

[30]:
```
shap.summary_plot(shap_values[5],X_train,feature_names = predictors)
```

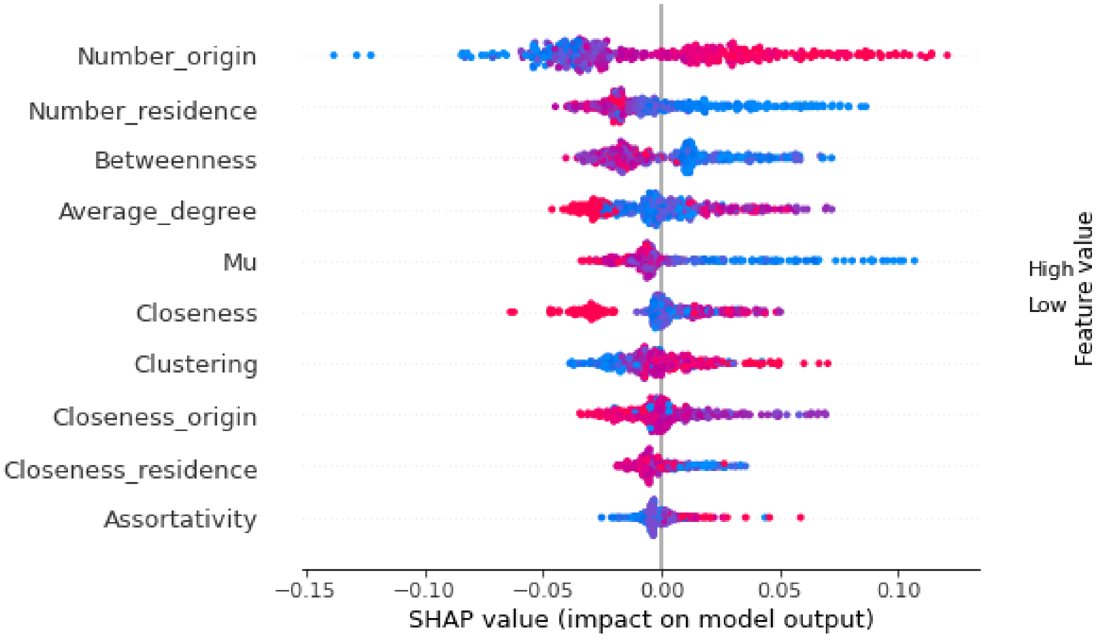

SHAP values for the control group

[31]:
```
shap.summary_plot(shap_values[0],X_train,feature_names = predictors)
```

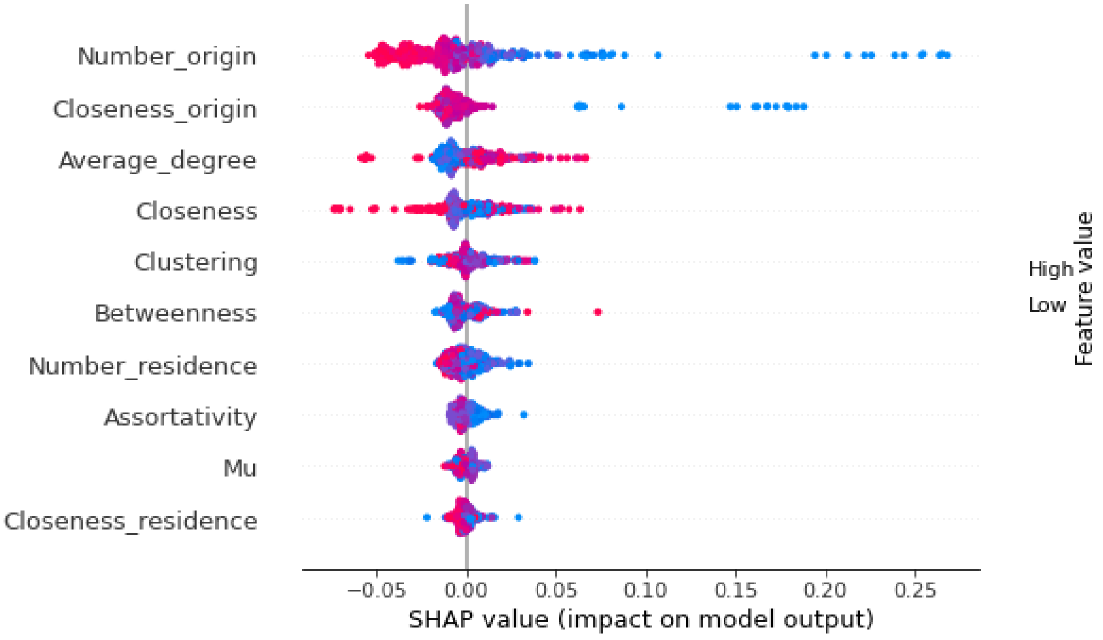

4 LIME

LIME (Local Interpretable Model-agnostic Explanations), is an algorithm that takes the decision function from the classifier (decision = f(features)). This function may be complex, but the algorithm makes a linear regression around a single prediction, weighting the importance of the coefficients with the distance to this local prediction.

This kind of algorithm helps us to explain single predictions.

[32]:
```
##Using LIME to interpret
import lime
import lime.lime_tabular
```

[33]:
```
explainer = lime.lime_tabular.LimeTabularExplainer(X_train,␣
↪feature_names=predictors, discretize_continuous=True)
```

[34]:
```
i = np.random.randint(0, X_test.shape[0])
exp = explainer.explain_instance(X_test[i], CV_rfc.predict_proba,␣
↪num_features=3, top_labels=1)
```

[35]:
```
exp.show_in_notebook(show_table=True, show_all=True)
```

```
<IPython.core.display.HTML object>
```

4.1 Artificial neural network

As a complementary method, we train a simple ANN to provide a new method and give more strength to the previous results. In order to do that, we will preprocess the data, distinguishing the categorical and numerical predictors. Then we will split the dataset into the train and test parts and, finally, we will define the model and fit to obtain a final result for the accuracy.

[36]:
```
### Import the package tensorflow
import os
os.environ['TF_CPP_MIN_LOG_LEVEL'] = '3'
import tensorflow as tf
import logging
logging.getLogger("tensorflow").setLevel(logging.ERROR)

tf.random.set_seed(0)
```

[40]:
```
###Define  a simple a ANN and fit our data
stat_accul = []
model_accul = tf.keras.Sequential([
    tf.keras.layers.Dense(70,activation="relu"),
    tf.keras.layers.Dense(70,activation="relu"),
    tf.keras.layers.Dense(6,activation="softmax")
])
```

```
###Compile the model
model_accul.compile(loss=tf.keras.losses.SparseCategoricalCrossentropy(),
              optimizer=tf.keras.optimizers.Adam(learning_rate=10e-4),
              metrics=["accuracy"])

### We fit the model 100 times and take notes of the accuracy on the test set

history_accul = model_accul.fit(X_train,
                          np.array(y_train),
                          epochs=100,
                          verbose = 0)
stat_accul.append(model_accul.evaluate(X_test,np.array(y_test))[1])
```

```
WARNING: AutoGraph could not transform <function
Model.make_train_function.<locals>.train_function at 0x7f2f8e7ae160> and will
run it as-is.
Please report this to the TensorFlow team. When filing the bug, set the
verbosity to 10 (on Linux, `export AUTOGRAPH_VERBOSITY=10`) and attach the full
output.
Cause: module 'gast' has no attribute 'Constant'
To silence this warning, decorate the function with
@tf.autograph.experimental.do_not_convert
WARNING: AutoGraph could not transform <function
Model.make_test_function.<locals>.test_function at 0x7f2f8d77c670> and will run
it as-is.
Please report this to the TensorFlow team. When filing the bug, set the
verbosity to 10 (on Linux, `export AUTOGRAPH_VERBOSITY=10`) and attach the full
output.
Cause: module 'gast' has no attribute 'Constant'
To silence this warning, decorate the function with
@tf.autograph.experimental.do_not_convert
3/3 [==============================] - 0s 1ms/step - loss: 1.7518 - accuracy:
0.4737
WARNING: AutoGraph could not transform <function
Model.make_train_function.<locals>.train_function at 0x7f2f8e7d2790> and will
run it as-is.
Please report this to the TensorFlow team. When filing the bug, set the
verbosity to 10 (on Linux, `export AUTOGRAPH_VERBOSITY=10`) and attach the full
output.
Cause: module 'gast' has no attribute 'Constant'
To silence this warning, decorate the function with
@tf.autograph.experimental.do_not_convert
WARNING: AutoGraph could not transform <function
Model.make_test_function.<locals>.test_function at 0x7f2f8c7710d0> and will run
it as-is.
Please report this to the TensorFlow team. When filing the bug, set the
```

```
verbosity to 10 (on Linux, `export AUTOGRAPH_VERBOSITY=10`) and attach the full
output.
Cause: module 'gast' has no attribute 'Constant'
To silence this warning, decorate the function with
@tf.autograph.experimental.do_not_convert
3/3 [==============================] - 0s 1ms/step - loss: 1.6754 - accuracy:
0.4526
WARNING: AutoGraph could not transform <function
Model.make_train_function.<locals>.train_function at 0x7f2f8e7905e0> and will
run it as-is.
Please report this to the TensorFlow team. When filing the bug, set the
verbosity to 10 (on Linux, `export AUTOGRAPH_VERBOSITY=10`) and attach the full
output.
Cause: module 'gast' has no attribute 'Constant'
To silence this warning, decorate the function with
@tf.autograph.experimental.do_not_convert
WARNING: AutoGraph could not transform <function
Model.make_test_function.<locals>.test_function at 0x7f2fb47d0dc0> and will run
it as-is.
Please report this to the TensorFlow team. When filing the bug, set the
verbosity to 10 (on Linux, `export AUTOGRAPH_VERBOSITY=10`) and attach the full
output.
Cause: module 'gast' has no attribute 'Constant'
To silence this warning, decorate the function with
@tf.autograph.experimental.do_not_convert
3/3 [==============================] - 0s 1ms/step - loss: 1.7216 - accuracy:
0.4526
WARNING: AutoGraph could not transform <function
Model.make_train_function.<locals>.train_function at 0x7f2fc8185160> and will
run it as-is.
Please report this to the TensorFlow team. When filing the bug, set the
verbosity to 10 (on Linux, `export AUTOGRAPH_VERBOSITY=10`) and attach the full
output.
Cause: module 'gast' has no attribute 'Constant'
To silence this warning, decorate the function with
@tf.autograph.experimental.do_not_convert
WARNING: AutoGraph could not transform <function
Model.make_test_function.<locals>.test_function at 0x7f2fc8185790> and will run
it as-is.
Please report this to the TensorFlow team. When filing the bug, set the
verbosity to 10 (on Linux, `export AUTOGRAPH_VERBOSITY=10`) and attach the full
output.
Cause: module 'gast' has no attribute 'Constant'
To silence this warning, decorate the function with
@tf.autograph.experimental.do_not_convert
3/3 [==============================] - 0s 1ms/step - loss: 1.7580 - accuracy:
0.4526
WARNING: AutoGraph could not transform <function
```

```
Model.make_train_function.<locals>.train_function at 0x7f2fb4257dc0> and will
run it as-is.
Please report this to the TensorFlow team. When filing the bug, set the
verbosity to 10 (on Linux, `export AUTOGRAPH_VERBOSITY=10`) and attach the full
output.
Cause: module 'gast' has no attribute 'Constant'
To silence this warning, decorate the function with
@tf.autograph.experimental.do_not_convert
WARNING: AutoGraph could not transform <function
Model.make_test_function.<locals>.test_function at 0x7f2f8dcd55e0> and will run
it as-is.
Please report this to the TensorFlow team. When filing the bug, set the
verbosity to 10 (on Linux, `export AUTOGRAPH_VERBOSITY=10`) and attach the full
output.
Cause: module 'gast' has no attribute 'Constant'
To silence this warning, decorate the function with
@tf.autograph.experimental.do_not_convert
3/3 [==============================] - 0s 1ms/step - loss: 1.7994 - accuracy:
0.4842
WARNING: AutoGraph could not transform <function
Model.make_train_function.<locals>.train_function at 0x7f2fb40e1940> and will
run it as-is.
Please report this to the TensorFlow team. When filing the bug, set the
verbosity to 10 (on Linux, `export AUTOGRAPH_VERBOSITY=10`) and attach the full
output.
Cause: module 'gast' has no attribute 'Constant'
To silence this warning, decorate the function with
@tf.autograph.experimental.do_not_convert
WARNING: AutoGraph could not transform <function
Model.make_test_function.<locals>.test_function at 0x7f2f8c7683a0> and will run
it as-is.
Please report this to the TensorFlow team. When filing the bug, set the
verbosity to 10 (on Linux, `export AUTOGRAPH_VERBOSITY=10`) and attach the full
output.
Cause: module 'gast' has no attribute 'Constant'
To silence this warning, decorate the function with
@tf.autograph.experimental.do_not_convert
3/3 [==============================] - 0s 1ms/step - loss: 1.7015 - accuracy:
0.4737
WARNING: AutoGraph could not transform <function
Model.make_train_function.<locals>.train_function at 0x7f2f8e779040> and will
run it as-is.
Please report this to the TensorFlow team. When filing the bug, set the
verbosity to 10 (on Linux, `export AUTOGRAPH_VERBOSITY=10`) and attach the full
output.
Cause: module 'gast' has no attribute 'Constant'
To silence this warning, decorate the function with
@tf.autograph.experimental.do_not_convert
```

```
WARNING: AutoGraph could not transform <function
Model.make_test_function.<locals>.test_function at 0x7f2f8e387310> and will run
it as-is.
Please report this to the TensorFlow team. When filing the bug, set the
verbosity to 10 (on Linux, `export AUTOGRAPH_VERBOSITY=10`) and attach the full
output.
Cause: module 'gast' has no attribute 'Constant'
To silence this warning, decorate the function with
@tf.autograph.experimental.do_not_convert
3/3 [==============================] - 0s 2ms/step - loss: 1.6747 - accuracy:
0.5368
WARNING: AutoGraph could not transform <function
Model.make_train_function.<locals>.train_function at 0x7f2fb40875e0> and will
run it as-is.
Please report this to the TensorFlow team. When filing the bug, set the
verbosity to 10 (on Linux, `export AUTOGRAPH_VERBOSITY=10`) and attach the full
output.
Cause: module 'gast' has no attribute 'Constant'
To silence this warning, decorate the function with
@tf.autograph.experimental.do_not_convert
WARNING: AutoGraph could not transform <function
Model.make_test_function.<locals>.test_function at 0x7f2f8c735940> and will run
it as-is.
Please report this to the TensorFlow team. When filing the bug, set the
verbosity to 10 (on Linux, `export AUTOGRAPH_VERBOSITY=10`) and attach the full
output.
Cause: module 'gast' has no attribute 'Constant'
To silence this warning, decorate the function with
@tf.autograph.experimental.do_not_convert
3/3 [==============================] - 0s 1ms/step - loss: 1.5728 - accuracy:
0.5053
WARNING: AutoGraph could not transform <function
Model.make_train_function.<locals>.train_function at 0x7f2f8e387280> and will
run it as-is.
Please report this to the TensorFlow team. When filing the bug, set the
verbosity to 10 (on Linux, `export AUTOGRAPH_VERBOSITY=10`) and attach the full
output.
Cause: module 'gast' has no attribute 'Constant'
To silence this warning, decorate the function with
@tf.autograph.experimental.do_not_convert
WARNING: AutoGraph could not transform <function
Model.make_test_function.<locals>.test_function at 0x7f2f8e09b040> and will run
it as-is.
Please report this to the TensorFlow team. When filing the bug, set the
verbosity to 10 (on Linux, `export AUTOGRAPH_VERBOSITY=10`) and attach the full
output.
Cause: module 'gast' has no attribute 'Constant'
To silence this warning, decorate the function with
```

```
@tf.autograph.experimental.do_not_convert
3/3 [==============================] - 0s 1ms/step - loss: 1.7425 - accuracy:
0.4842
WARNING: AutoGraph could not transform <function
Model.make_train_function.<locals>.train_function at 0x7f2f8e608310> and will
run it as-is.
Please report this to the TensorFlow team. When filing the bug, set the
verbosity to 10 (on Linux, `export AUTOGRAPH_VERBOSITY=10`) and attach the full
output.
Cause: module 'gast' has no attribute 'Constant'
To silence this warning, decorate the function with
@tf.autograph.experimental.do_not_convert
WARNING: AutoGraph could not transform <function
Model.make_test_function.<locals>.test_function at 0x7f2fb4257280> and will run
it as-is.
Please report this to the TensorFlow team. When filing the bug, set the
verbosity to 10 (on Linux, `export AUTOGRAPH_VERBOSITY=10`) and attach the full
output.
Cause: module 'gast' has no attribute 'Constant'
To silence this warning, decorate the function with
@tf.autograph.experimental.do_not_convert
3/3 [==============================] - 0s 2ms/step - loss: 1.6828 - accuracy:
0.5053
```

4.2 Display the final results

[41]:
```
print(f"The final results for a training iteration is {np.average(stat_accul):.
 ↪2f}")
```

```
The final results for a training iteration is 0.48
```

[ ]:
```
```
