# Supplementary material for: Structural measures of personal networks predict migrants’ cultural backgrounds: an explanation from Grid/Group theory
Source: PNAS Nexus. 2022 Sep 21;1(4):pgac195. doi: 10.1093/pnasnexus/pgac195 (PMC9802079; doi:10.1093/pnasnexus/pgac195)
Supplement: pgac195_Supplemental_Files [file pgac195_supplemental_files.zip › PNASNEXUS-PNASNEXUS-2022-00039-T-s05.pdf]

Analysis-RELG

December 30, 2021

1 Analysis

In this notebook we will take the data from the *Ego networks* notebook and make an analysis with the three different methods: a multinomial logistic model, a random forest method an an artificial neural network. First, we will load the data, we will check for outliers and then we will prepare and format the predictors in order to apply each one of these methods. The first step is loading the libraries, in this case we will use the standard numpy, pandas, matplotlib and seaborn for manipulating and plotting the data. In order to apply the different techniques of analysis, we will use sklearn, statsmodels and tensorflow.

[1]:
```
import pandas as pd
import matplotlib.pyplot as plt
import numpy as np
import seaborn as sns
# Sklearn
from sklearn.ensemble import RandomForestClassifier
from sklearn.model_selection import train_test_split, cross_validate,␣
 ↪cross_val_predict
from sklearn.metrics import classification_report, confusion_matrix,␣
 ↪accuracy_score
from sklearn.dummy import DummyClassifier
from sklearn.preprocessing import StandardScaler
from sklearn.model_selection import GridSearchCV
# Statsmodels
import statsmodels.formula.api as smf
from statsmodels.api import MNLogit




# Just to print prettier. Uncomment to see all (not important) warnings
import warnings
warnings.filterwarnings('ignore')

%matplotlib inline
```

1.1 Load data

The next step is loading the .csv file from the previous notebook. Then we will select the columns we will use for the analysis, as the notebook contains a lot of information of the egos not related to the structural properties of their networks. Then we will map the categorical columns to a numerical encoding in the columns of : *Subject origin*, *Subject residence*, and *Regime*.

[2]:
```
### Read data
df_2 = pd.read_csv('Redes_2_relg.csv')
### Drop Unnecessary Variables
df_2.drop('Unnamed: 0',axis=1, inplace=True)

###Take the necessary ones
df = df_2[df_2.columns[0:30]]
df['EDUC'] = df_2['EDUC'].copy()
df['FMIG2'] = df_2['FMIG2'].copy()
df['SEX'] = df_2['SEX'].copy()
df['RELG'] = df_2['RELG'].copy()

### The numerical encoding
#not_apply = ['Subject_origin','Subject_residence','Regime']
not_apply = ['Subject_origin','Subject_residence']
diccs = [0]*len(not_apply)
i = 0
for col in not_apply:
        uniques = list(df[col].unique())
        diccs[i] = {uniques[j]:uniques.index(uniques[j]) for j in␣
 ↪range(len(uniques)) }
        df[col] = df[col].map(diccs[i])
        i+=1
df.columns = df.columns.str.replace(' ', '_')
### Reset the datatype of the columns
df['Subject_origin'].astype('int64')
df['Subject_residence'].astype('int64')
#df['Regime'].astype('int64')
```

[2]:
```
0      0
1      0
2      0
3      0
4      0
      ..
468    1
469    1
470    1
471    1
472    1
Name: Subject_residence, Length: 473, dtype: int64
```

1.2 Prepare and explore data

We make an overview of the main statistics of the data and the properties we have generated in the past notebook.

[3]:
```
df.describe(include='all')
```

[3]:
```
       Subject_origin  Subject_residence          Mu   Regime  \
count      473.000000         473.000000  473.000000      473
unique            NaN                NaN         NaN        3
top               NaN                NaN         NaN  Unclear
freq              NaN                NaN         NaN      222
mean         4.997886           0.596195   -0.743170      NaN
std          2.781978           0.491179   13.524199      NaN
min          0.000000           0.000000 -294.081935      NaN
25%          2.000000           0.000000   -0.299994      NaN
50%          5.000000           1.000000   -0.111711      NaN
75%          8.000000           1.000000    0.100436      NaN
max          9.000000           1.000000    2.302179      NaN

       Average_degree  Betweenness   Closeness  Load_centrality  \
count      473.000000   473.000000  473.000000       473.000000
unique            NaN          NaN         NaN              NaN
top               NaN          NaN         NaN              NaN
freq              NaN          NaN         NaN              NaN
mean        23.921957     0.015319    0.687610         0.014895
std         13.589893     0.011304    0.217223         0.011652
min          2.628571     0.000000    0.130665         0.000000
25%         12.818182     0.006953    0.532497         0.005732
50%         19.066667     0.013948    0.632030         0.013860
75%         40.666667     0.020487    0.938077         0.020487
max         44.000000     0.078431    1.000000         0.078431

       Assortativity  Clustering  …        Asmo        Arac        Asex  \
count     473.000000  473.000000  …  473.000000  473.000000  473.000000
unique           NaN         NaN  …         NaN         NaN         NaN
top              NaN         NaN  …         NaN         NaN         NaN
freq             NaN         NaN  …         NaN         NaN         NaN
mean       -0.022917    0.640939  …   -0.088291   -0.153129   -0.009537
std         0.231357    0.168242  …    0.417944    0.577599    0.149704
min        -2.000000    0.206979  …   -2.000000   -2.000000   -2.000000
25%        -0.136488    0.506595  …   -0.032954   -0.037886   -0.029268
50%        -0.022727    0.661885  …   -0.022727   -0.022727   -0.022727
75%         0.018041    0.771862  …    0.006434    0.004354    0.013147
max         0.974478    1.000000  …    0.496721    1.000000    0.597828

       EGOFIRST  EGOLAST         SEX        BORN        EDUC       FMIG2  \
count       473      473  473.000000  473.000000  473.000000  473.000000
```

```
unique      369    359        NaN        NaN        NaN        NaN
top        jose  perez        NaN        NaN        NaN        NaN
freq         13      7        NaN        NaN        NaN        NaN
mean        NaN    NaN   1.448203   3.014799   3.509514   3.634249
std         NaN    NaN   0.497836   1.460567   1.432231   6.618497
min         NaN    NaN   1.000000   1.000000   1.000000  -1.000000
25%         NaN    NaN   1.000000   2.000000   2.000000   0.000000
50%         NaN    NaN   1.000000   2.000000   4.000000   1.000000
75%         NaN    NaN   2.000000   4.000000   4.000000   4.000000
max         NaN    NaN   2.000000   7.000000   7.000000  45.000000

              RELG
count   473.000000
unique         NaN
top            NaN
freq           NaN
mean      2.080338
std       0.665345
min       1.000000
25%       2.000000
50%       2.000000
75%       3.000000
max       3.000000

[11 rows x 33 columns]
```

Some values of mu are way out of range (min = -294). This is clearly from divergences in the model. We mark observations greater than 10 (in absolute value) as nan and then drop nan.

[4]:
```
# Clean estimates for mu
df['Mu'] = df['Mu'].apply(lambda x: np.nan if x < -100 else x)
df['Mu'] = df['Mu'].apply(lambda x: np.nan if x > 100 else x)
df.dropna(inplace = True)
```

[5]:
```
dicc_final = {1:"Other",2:"Christian",3:"Muslim"}
```

1.2.1 Define predictors for all the inference and prediction methods

[6]:
```
predictors =␣
↪['Closeness','Clustering','Average_degree','Assortativity','Betweenness',
           ␣
↪'Closeness_origin','Closeness_residence','Number_origin','Number_residence','Mu',
             'Afrq','Aol2','Apro','Arel','Clos','Arac','Asex']
target = "RELG"
```

1.2.2 Define train and test split for the dataset

[7]:
```
X = df[predictors]        # independent variables
y = df[target]

test_size = 0.20 #maybe more is needed (20% is standard though)
X_train, X_test, y_train, y_test = train_test_split(X, y, test_size =␣
 ↪test_size, random_state = 0)


# Standard Scaler
sc = StandardScaler()
X_train = sc.fit_transform(X_train)
X_test = sc.transform (X_test)

#Define dataframe as merge of X and y
df_str = df[target].to_frame().merge(pd.DataFrame(sc.
 ↪fit_transform(X),columns=predictors,index=df.index), left_index=True,␣
 ↪right_index=True)
```

2 INFERENCE

At this point, we begin to include tools of inference, beginning by the multinomial logistic regression (MLN). The library used for this analysis is mainly *statsmodels* and the main function can be checked in this link: https://stats.idre.ucla.edu/stata/dae/multinomiallogistic-regression/

In this part of the notebook we will prepare the variables, execute the regression and save the results.

2.0.1 Fit Multinomial Logistic Model

https://www.statsmodels.org/stable/generated/statsmodels.discrete.discrete_model.MNLogit.html

[8]:
```
### Uses the list 'predictors' as independent variables
formula_predictors = ' + '.join(predictors)
target_str = target +" ~ {}"
model = MNLogit.from_formula(target_str.format(formula_predictors), df_str)
results = model.fit(maxiter=200)
```

```
Optimization terminated successfully.
         Current function value: 0.800972
         Iterations 7
```

Results

[9]:
```
print(results.summary())
```

```
                          MNLogit Regression Results
==============================================================================
```

Dep. Variable:	RELG	No. Observations:	472
Model:	MNLogit	Df Residuals:	436
Method:	MLE	Df Model:	34
Date:	Thu, 30 Dec 2021	Pseudo R-squ.:	0.1913
Time:	04:26:04	Log-Likelihood:	-378.06
converged:	True	LL-Null:	-467.51
Covariance Type:	nonrobust	LLR p-value:	1.389e-21

RELG=2	coef	std err	z	P>|z|	[0.025	0.975]
Intercept	1.5445	0.175	8.845	0.000	1.202	1.887
Closeness	-0.4742	0.958	-0.495	0.621	-2.353	1.404
Clustering	0.2209	0.169	1.308	0.191	-0.110	0.552
Average_degree	0.9179	0.890	1.031	0.302	-0.827	2.662
Assortativity	0.0347	0.179	0.194	0.846	-0.316	0.385
Betweenness	0.6419	0.272	2.363	0.018	0.109	1.174
Closeness_origin	-0.3801	0.164	-2.319	0.020	-0.701	-0.059
Closeness_residence	-0.2125	0.217	-0.980	0.327	-0.637	0.212
Number_origin	0.6460	0.253	2.558	0.011	0.151	1.141
Number_residence	-0.0354	0.197	-0.180	0.857	-0.422	0.351
Mu	-0.1672	0.211	-0.793	0.428	-0.581	0.246
Afrq	-0.2488	0.204	-1.221	0.222	-0.648	0.151
Aol2	-0.1818	0.149	-1.218	0.223	-0.474	0.111
Apro	-1.3684	0.409	-3.342	0.001	-2.171	-0.566
Arel	-0.0073	0.226	-0.032	0.974	-0.450	0.436
Clos	0.6494	0.291	2.231	0.026	0.079	1.220
Arac	-0.0481	0.193	-0.250	0.803	-0.426	0.330
Asex	-0.0419	0.240	-0.175	0.861	-0.512	

```
0.428
--------------------------------------------------------------------------------
-------
          RELG=3       coef    std err          z      P>|z|      [0.025
0.975]
--------------------------------------------------------------------------------
-------
Intercept              0.2287      0.227      1.006      0.314      -0.217
0.674
Closeness             -1.1744      1.074     -1.093      0.274      -3.280
0.931
Clustering             0.5847      0.208      2.815      0.005       0.178
0.992
Average_degree         0.8057      1.024      0.787      0.431      -1.201
2.812
Assortativity          0.0910      0.210      0.433      0.665      -0.321
0.503
Betweenness            0.1294      0.345      0.375      0.708      -0.547
0.806
Closeness_origin      -0.5432      0.269     -2.017      0.044      -1.071
-0.015
Closeness_residence   -0.1964      0.235     -0.836      0.403      -0.657
0.264
Number_origin          2.1877      0.491      4.456      0.000       1.225
3.150
Number_residence       1.1059      0.401      2.758      0.006       0.320
1.892
Mu                    -0.0880      0.235     -0.375      0.707      -0.548
0.372
Afrq                   0.0858      0.232      0.370      0.711      -0.368
0.540
Aol2                  -0.5346      0.200     -2.673      0.008      -0.927
-0.143
Apro                  -1.3338      0.424     -3.148      0.002      -2.164
-0.503
Arel                  -0.4734      0.277     -1.708      0.088      -1.016
0.070
Clos                   0.4877      0.307      1.590      0.112      -0.113
1.089
Arac                  -0.2360      0.196     -1.205      0.228      -0.620
0.148
Asex                   1.2337      0.328      3.761      0.000       0.591
1.877
================================================================================
=======
```

[10]:
```
print('pseudo r-squared = {}'.format(np.round(results.prsquared,2)))
```

```
pseudo r-squared = 0.19
```

```
[11]: results.llr_pvalue
```

```
[11]: 1.3889296381166788e-21
```

3 PREDICTION

We train and fit a powerful non-linear (and non-parametric) machine learnin classifier to the data; a Random Forest. There are many other alternatives, but tree based metods are very powerfull and there are new techniques to help identify relevant predictors.

In this section, we want to test wether this model can outperform significantly other null (dummy) classifiers. If that is the case (which it is), it confirms the hypothesis that the predictors have relevant information about the nationalities of the subjects.

3.0.1 Train and test with MNL regression

```
[12]: formula_predictors = ' + '.join(predictors)
model = MNLogit.from_formula(target_str.format(formula_predictors), df_str.
 ↪loc[y_train.index])
results_prediction = model.fit(maxiter=200)
ypred = results_prediction.predict(df_str.loc[y_test.index])
y_pred =list(map(np.argmax,np.array(ypred)))
##Meter función accuracy
```

```
Optimization terminated successfully.
         Current function value: 0.773547
         Iterations 7
```

```
[13]: from sklearn.metrics import accuracy_score
print(accuracy_score(y_test, y_pred))
```

```
0.23157894736842105
```

3.0.2 Train and tune the model using k-cross fold validation

```
[14]: scoring = 'accuracy' #'f1_macro' # This chooses the metric to optimise during␣
 ↪training (there are others!)
njobs=-1                          # This the number of cores used in your cpu␣
 ↪(-1 means "all of them")
cv=5                              # the k in k-cross-fold validation
# RANDOM FOREST
print('\nFitting Random Forest\n')

rfc=RandomForestClassifier(random_state=0)
# Parameter combinations to explore
```

```
param_grid = {
    'n_estimators': [75, 100,300,1000],
    'max_features': ['auto', None],
    'min_samples_split' :[2,6, 10, 14],
    'max_depth' : [10, 15, 30, 50,None],
    'max_samples' : [0.5 ,0.7, None],}


CV_rfc = GridSearchCV(estimator=rfc,
                      param_grid=param_grid,
                      scoring = scoring,
                      verbose=0,
                      n_jobs=njobs,
                      cv= cv)
CV_rfc.fit(X_train, y_train)

print('\nRandom Forest:')
print('Best Score: ', CV_rfc.best_score_)
print('Best Params: ', CV_rfc.best_params_)
```

```
Fitting Random Forest


Random Forest:
Best Score:  0.6367368421052632
Best Params:  {'max_depth': 10, 'max_features': None, 'max_samples': 0.5,
'min_samples_split': 10, 'n_estimators': 100}
```

3.0.3 Evaluating the algorithm performance in the test set (unseen data)

[15]:
```
y_pred = CV_rfc.predict(X_test)
print('Confusion Matrix:\n ', confusion_matrix(y_test,y_pred),'\n')
print(classification_report(y_test,y_pred),'\n')
print('Accuracy: {0:.2f}'.format(accuracy_score(y_test, y_pred),2))
```

```
Confusion Matrix:
  [[ 2 15  2]
 [ 2 43  5]
 [ 0 15 11]]

              precision    recall  f1-score   support

           1       0.50      0.11      0.17        19
           2       0.59      0.86      0.70        50
           3       0.61      0.42      0.50        26
```

```
    accuracy                           0.59        95
   macro avg       0.57      0.46      0.46        95
weighted avg       0.58      0.59      0.54        95
```

```
Accuracy: 0.59
```

3.0.4 Compare this performance with null models

[16]:
```
#  relative prevalence of each class
rel_prev = (y.value_counts() / len(y))
print(rel_prev)
```

```
2    0.552966
3    0.262712
1    0.184322
Name: RELG, dtype: float64
```

[17]:
```
# Uniform Dummy Classifier (classifies randomly with p = 1/6)

# If the classifier randomly guesses:
print('Acurracy of uniform dummy classifier: ',(((1/6) * y.value_counts()) /␣
 ↪len(y)).sum()) # = 1/6
```

```
Acurracy of uniform dummy classifier:  0.16666666666666666
```

[18]:
```
# Stratified Dummy Classifier (classifies randomly with p ~ prevalence of each␣
 ↪class)
print('Acurracy of stratified dummy classifier: ',(rel_prev * y.value_counts()).
 ↪sum() / len(y))
```

```
Acurracy of stratified dummy classifier:  0.4087636455041655
```

[19]:
```
# Most frequent Dummy Classifier (classifies always in the most frequent class)
print('Acurracy of Most freq dummy classifier: ',rel_prev.max() )
```

```
Acurracy of Most freq dummy classifier:  0.5529661016949152
```

[20]:
```
# SKLEARN versions of the dummy classifiers (to double check and for␣
 ↪convinience methods)

dummy = "stratified"# most_frequent, stratified, uniform
dummy_clf = DummyClassifier(strategy=dummy,random_state=0)




# Actual accuracy of the dummy in the same train-test split as the RF model
dummy_clf.fit(X_train, y_train)
```

```
dummy_score = dummy_clf.score(X_test, y_test)
print('Mean accuracy of null ' + dummy +' model: {0:.2f}'.
 ↪format(dummy_score),'\n')
print('Mean accuracy (in test) of RF model: {0:.2f}'.format(CV_rfc.
 ↪score(X_test, y_test)),'\n')
```

```
Mean accuracy of null stratified model: 0.39

Mean accuracy (in test) of RF model: 0.59
```

[21]:
```
# Confusion matrix and report of the selected dummy classifier

y_pred_dummy = dummy_clf.predict(X_test)
print('Confusion Matrix:\n\n ',confusion_matrix(y_test,y_pred_dummy),'\n')
print(classification_report(y_test,y_pred_dummy),'\n')
print('Accuracy: {0:.2f}'.format(accuracy_score(y_test, y_pred_dummy),2))
```

```
Confusion Matrix:

  [[ 3 12  4]
 [ 6 27 17]
 [ 3 16  7]]

              precision    recall  f1-score   support

           1       0.25      0.16      0.19        19
           2       0.49      0.54      0.51        50
           3       0.25      0.27      0.26        26

    accuracy                           0.39        95
   macro avg       0.33      0.32      0.32        95
weighted avg       0.38      0.39      0.38        95


Accuracy: 0.39
```

[22]:
```
# Just for reference, the results of the RF Model

y_pred = CV_rfc.predict(X_test)
print('Confusion Matrix:\n\n ', confusion_matrix(y_test,y_pred),'\n')
print(classification_report(y_test,y_pred),'\n')
print('Accuracy: {0:.2f}'.format(accuracy_score(y_test, y_pred),2))
```

```
Confusion Matrix:

  [[ 2 15  2]
 [ 2 43  5]
```

```
[ 0 15 11]]

              precision    recall  f1-score   support

           1       0.50      0.11      0.17        19
           2       0.59      0.86      0.70        50
           3       0.61      0.42      0.50        26

    accuracy                           0.59        95
   macro avg       0.57      0.46      0.46        95
weighted avg       0.58      0.59      0.54        95


Accuracy: 0.59
```

[23]:
```
dummy_report = pd.DataFrame(classification_report(y_test,dummy_clf.
 ↪predict(X_test), output_dict= True))

rfc_report = pd.DataFrame(classification_report(y_test,CV_rfc.predict(X_test),␣
 ↪output_dict= True))
```

Increase in prediction power (percentage with respect to null model) i.e. 100% means twice as good

[24]:
```
final_table = ((rfc_report - dummy_report)*100 / dummy_report).drop('support').
 ↪round(decimals=2)
final_table
```

[24]:

	1	2	3	accuracy	macro avg	weighted avg
precision	100.00	19.99	144.44	51.35	71.57	53.21
recall	-33.33	59.26	57.14	51.35	43.55	51.35
f1-score	-10.14	35.95	92.86	51.35	41.98	41.88

This significant increases further support the claim that the predictors (based on ego-network properties) have useful information to predict the countries of origin of the individuals)

3.1 Shap Values

Shap values are a tool to interpret our random forest model, in this case. They tell us some intuition about which part of the prediction belongs to each feature.

A positive (negative) SHAP value indicates that the value (in this case, probability of belonging to a certain country) is reinforced (diminished) by the feature.

We will use 2 kind of plots at this moment. The first one one is a summary plot, a violin plot of the distribution of SHAP values. The colour indicates the value of the feature indicated at the left. This plot let us see the which features contribute the most (this is, they have high SHAP values). Features are ordered according to their contribution to the global prediction.

The second kind of plot you will see several times after the summary plot is the dependence plot. They show the distribution of the SHAP values of a variable. The colormap plots another variable, the one the algorithm thinks it has more interaction with the current variable. It lets us distinguish between different regimes of the coloured variable.

[25]:
```
# explain the model's predictions using SHAP
##Shap values
import  shap

shap.initjs()
model = CV_rfc.best_estimator_
explainer = shap.TreeExplainer(model,X_train,check_additivity=False)
shap_values = explainer.shap_values(X_train,check_additivity=False)
```

```
<IPython.core.display.HTML object>
```

3.2 Example of summary plot

We extract the summary plots that summarizes the correlations for each nationality.

SHAP values for the control group

[26]:
```
shap.summary_plot(shap_values[0],X_train,feature_names = predictors)
```

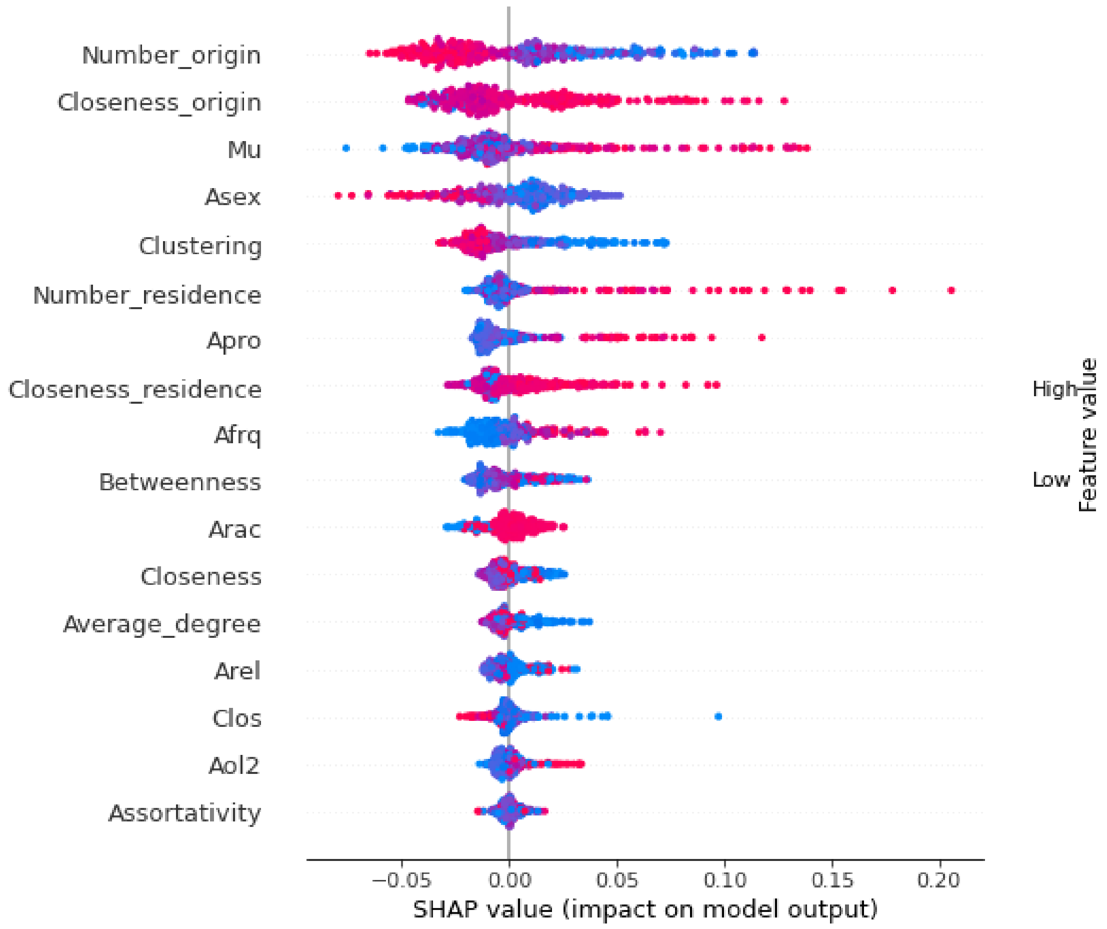

SHAP values for the christians

[27]:
```
shap.summary_plot(shap_values[1],X_train,feature_names = predictors)
```

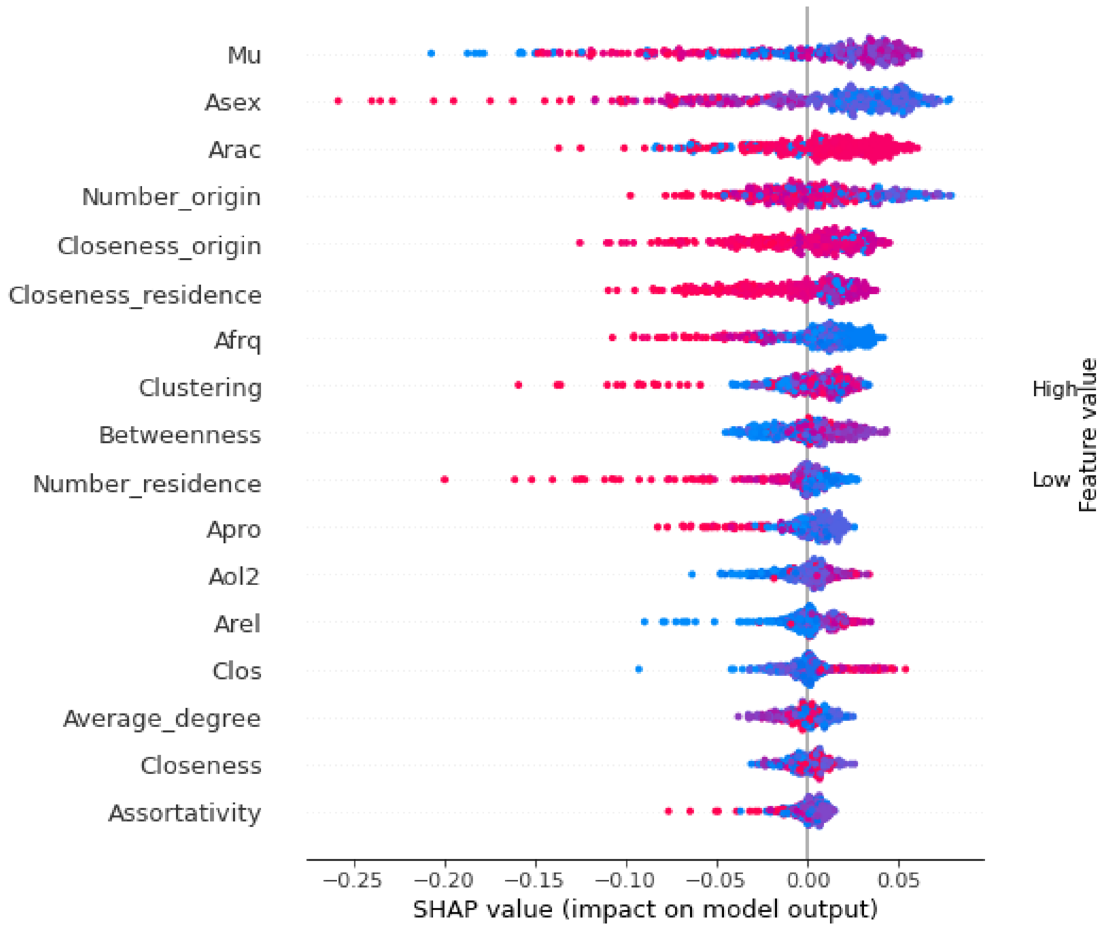

SHAP values for the muslims

[28]:
```
shap.summary_plot(shap_values[2],X_train,feature_names = predictors)
```

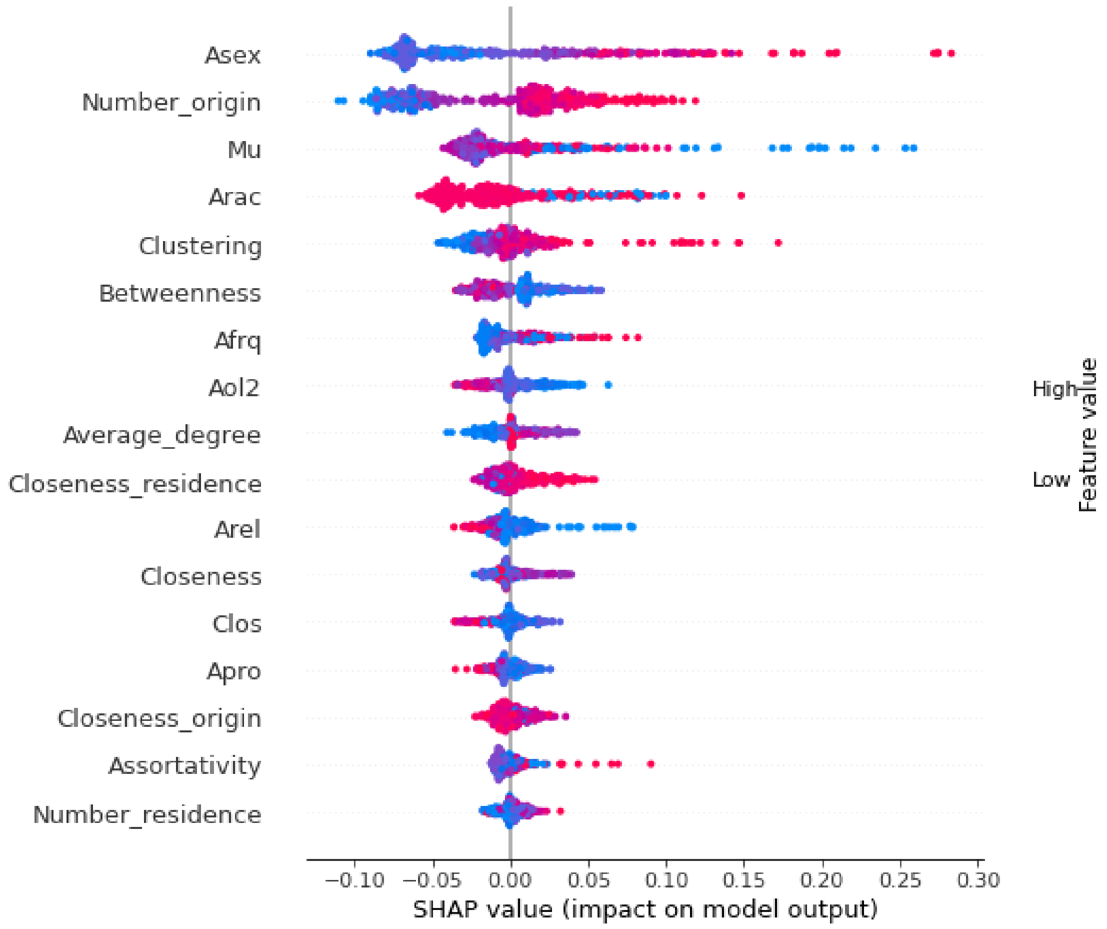

4 LIME

LIME (Local Interpretable Model-agnostic Explanations), is an algorithm that takes the decision function from the classifier (decision = f(features)). This function may be complex, but the algorithm makes a linear regression around a single prediction, weighting the importance of the coefficients with the distance to this local prediction.

This kind of algorithm helps us to explain single predictions.

[29]:
```
##Using LIME to interpret
import lime
import lime.lime_tabular
```

[30]:
```
explainer = lime.lime_tabular.LimeTabularExplainer(X_train,␣
 ↪feature_names=predictors, discretize_continuous=True)
```

[31]:
```
i = np.random.randint(0, X_test.shape[0])
```

```
exp = explainer.explain_instance(X_test[i], CV_rfc.predict_proba,␣
↪num_features=3, top_labels=1)
```

[32]:
```
exp.show_in_notebook(show_table=True, show_all=True)
```

<IPython.core.display.HTML object>

4.1 Artificial neural network

As a complementary method, we train a simple ANN to provide a new method and give more strength to the previous results. In order to do that, we will preprocess the data, distinguishing the categorical and numerical predictors. Then we will split the dataset into the train and test parts and, finally, we will define the model and fit to obtain a final result for the accuracy.

[33]:
```
### Import the package tensorflow
import os
os.environ['TF_CPP_MIN_LOG_LEVEL'] = '3'
import logging
logging.getLogger("tensorflow").setLevel(logging.ERROR)
import tensorflow as tf

tf.random.set_seed(0)
```

[37]:
```
###Define  a simple a ANN and fit our data
stat_accul = []
model_accul = tf.keras.Sequential([
    tf.keras.layers.Dense(70,activation="relu"),
    tf.keras.layers.Dense(70,activation="relu"),
    tf.keras.layers.Dense(70,activation="relu"),
    tf.keras.layers.Dense(4,activation="softmax")
])



###Compile the model
model_accul.compile(loss=tf.keras.losses.SparseCategoricalCrossentropy(),
               optimizer=tf.keras.optimizers.Adam(learning_rate=10e-4),
               metrics=["accuracy"])

### We fit the model 100 times and take notes of the accuracy on the test set

history_accul = model_accul.fit(X_train,
                         np.array(y_train),
                         epochs=100,
                         verbose = 0)
stat_accul.append(model_accul.evaluate(X_test,np.array(y_test))[1])
```

```
WARNING:tensorflow:AutoGraph could not transform <function
Model.make_train_function.<locals>.train_function at 0x7fcd152de9d0> and will
run it as-is.
Please report this to the TensorFlow team. When filing the bug, set the
verbosity to 10 (on Linux, `export AUTOGRAPH_VERBOSITY=10`) and attach the full
output.
Cause: module 'gast' has no attribute 'Constant'
To silence this warning, decorate the function with
@tf.autograph.experimental.do_not_convert
WARNING: AutoGraph could not transform <function
Model.make_train_function.<locals>.train_function at 0x7fcd152de9d0> and will
run it as-is.
Please report this to the TensorFlow team. When filing the bug, set the
verbosity to 10 (on Linux, `export AUTOGRAPH_VERBOSITY=10`) and attach the full
output.
Cause: module 'gast' has no attribute 'Constant'
To silence this warning, decorate the function with
@tf.autograph.experimental.do_not_convert
WARNING:tensorflow:AutoGraph could not transform <function
Model.make_test_function.<locals>.test_function at 0x7fcd15268430> and will run
it as-is.
Please report this to the TensorFlow team. When filing the bug, set the
verbosity to 10 (on Linux, `export AUTOGRAPH_VERBOSITY=10`) and attach the full
output.
Cause: module 'gast' has no attribute 'Constant'
To silence this warning, decorate the function with
@tf.autograph.experimental.do_not_convert
WARNING: AutoGraph could not transform <function
Model.make_test_function.<locals>.test_function at 0x7fcd15268430> and will run
it as-is.
Please report this to the TensorFlow team. When filing the bug, set the
verbosity to 10 (on Linux, `export AUTOGRAPH_VERBOSITY=10`) and attach the full
output.
Cause: module 'gast' has no attribute 'Constant'
To silence this warning, decorate the function with
@tf.autograph.experimental.do_not_convert
3/3 [==============================] - 0s 1ms/step - loss: 2.4842 - accuracy:
0.5474
```

4.2 Display the final results

[38]:
```
print(f"The final results for a training iteration is {np.average(stat_accul):.
 ↪2f}")
```

```
The final results for a training iteration is 0.55
```

[ ]:
```

```
