# Supplementary material for: Structural measures of personal networks predict migrants’ cultural backgrounds: an explanation from Grid/Group theory
Source: PNAS Nexus. 2022 Sep 21;1(4):pgac195. doi: 10.1093/pnasnexus/pgac195 (PMC9802079; doi:10.1093/pnasnexus/pgac195)
Supplement: pgac195_Supplemental_Files [file pgac195_supplemental_files.zip › PNASNEXUS-PNASNEXUS-2022-00039-T-s07.pdf]

Analysis_dominican_version

June 9, 2022

1 Analysis

In this notebook we will take the data from the *Ego networks* notebook and make an analysis with the three different methods: a multinomial logistic model, a random forest method an an artificial neural network. First, we will load the data, we will check for outliers and then we will prepare and format the predictors in order to apply each one of these methods. The first step is loading the libraries, in this case we will use the standard numpy, pandas, matplotlib and seaborn for manipulating and plotting the data. In order to apply the different techniques of analysis, we will use sklearn, statsmodels and tensorflow.

[1]:
```
import pandas as pd
import matplotlib.pyplot as plt
import numpy as np
import seaborn as sns
# Sklearn
from sklearn.ensemble import RandomForestClassifier
from sklearn.model_selection import train_test_split, cross_validate,␣
↪cross_val_predict
from sklearn.metrics import classification_report, confusion_matrix,␣
↪accuracy_score
from sklearn.dummy import DummyClassifier
from sklearn.preprocessing import StandardScaler
from sklearn.model_selection import GridSearchCV
# Statsmodels
import statsmodels.formula.api as smf
from statsmodels.api import MNLogit



# Just to print prettier. Uncomment to see all (not important) warnings
import warnings
warnings.filterwarnings('ignore')

%matplotlib inline
```

1.1 Load data

The next step is loading the .csv file from the previous notebook. Then we will select the columns we will use for the analysis, as the notebook contains a lot of information of the egos not related to the structural properties of their networks. Then we will map the categorical columns to a numerical encoding in the columns of : *Subject origin*, *Subject residence*, and *Regime*.

[2]:
```
### Read data
df_2 = pd.read_csv('Redes_2.csv')
### Drop Unnecessary Variables
df_2.drop('Unnamed: 0',axis=1, inplace=True)

###Take the necessary ones
df = df_2[df_2.columns[0:17]]
df['EDUC'] = df_2['EDUC'].copy()
df['FMIG2'] = df_2['FMIG2'].copy()
df['SEX'] = df_2['SEX'].copy()
df['RELG'] = df_2['RELG'].copy()

### The numerical encoding
#not_apply = ['Subject_origin','Subject_residence','Regime']
not_apply = ['Subject_origin','Subject_residence']
diccs = [0]*len(not_apply)
i = 0
for col in not_apply:
        uniques = list(df[col].unique())
        diccs[i] = {uniques[j]:uniques.index(uniques[j]) for j in␣
 ↪range(len(uniques)) }
        df[col] = df[col].map(diccs[i])
        i+=1
df.columns = df.columns.str.replace(' ', '_')
### Reset the datatype of the columns
df['Subject_origin'].astype('int64')
df['Subject_residence'].astype('int64')
#df['Regime'].astype('int64')
df.dropna(inplace=True)
```

1.2 Prepare and explore data

We make an overview of the main statistics of the data and the properties we have generated in the past notebook.

[3]:
```
df.describe(include='all')
```

[3]:

	Subject_num	Subject_origin	Subject_residence	Mu	Regime \
count	473.000000	473.000000	473.000000	473.000000	473
unique	NaN	NaN	NaN	NaN	3
top	NaN	NaN	NaN	NaN	Unclear

freq	NaN	NaN	NaN	NaN	222
mean	48.919662	4.997886	0.596195	-0.743170	NaN
std	39.256840	2.781978	0.491179	13.524199	NaN
min	1.000000	0.000000	0.000000	-294.081935	NaN
25%	17.000000	2.000000	0.000000	-0.299994	NaN
50%	42.000000	5.000000	1.000000	-0.111711	NaN
75%	66.000000	8.000000	1.000000	0.100436	NaN
max	161.000000	9.000000	1.000000	2.302179	NaN

	Average_degree	Betweenness	Closeness	Load_centrality	\
count	473.000000	473.000000	473.000000	473.000000	
unique	NaN	NaN	NaN	NaN	
top	NaN	NaN	NaN	NaN	
freq	NaN	NaN	NaN	NaN	
mean	23.910303	0.015374	0.687343	0.014964	
std	13.574373	0.011329	0.216891	0.011674	
min	2.628571	0.000023	0.130665	0.000023	
25%	12.818182	0.006953	0.532497	0.005732	
50%	19.066667	0.014024	0.632030	0.013907	
75%	40.666667	0.020487	0.938077	0.020487	
max	43.955556	0.078431	0.999012	0.078431	

	Assortativity	…	Number_components	Size_largest_component	\
count	473.000000	…	473.000000	473.000000	
unique	NaN	…	NaN	NaN	
top	NaN	…	NaN	NaN	
freq	NaN	…	NaN	NaN	
mean	-0.028397	…	1.162791	0.985243	
std	0.213720	…	0.496737	0.053987	
min	-0.695654	…	1.000000	0.500000	
25%	-0.139165	…	1.000000	1.000000	
50%	-0.045455	…	1.000000	1.000000	
75%	0.024567	…	1.000000	1.000000	
max	0.974478	…	6.000000	1.000000	

	Closeness_residence	Number_residence	Closeness_origin	\
count	473.000000	473.000000	473.000000	
unique	NaN	NaN	NaN	
top	NaN	NaN	NaN	
freq	NaN	NaN	NaN	
mean	2.766545	12.562368	2.495494	
std	1.232411	10.960871	0.876297	
min	0.000000	0.000000	0.000000	
25%	2.263158	4.000000	2.062500	
50%	3.000000	10.000000	2.560976	
75%	3.600000	19.000000	3.052632	
max	5.000000	66.000000	4.883721	

```
        Number_origin        EDUC        FMIG2         SEX        RELG
count      473.000000  473.000000   473.000000  473.000000  473.000000
unique            NaN         NaN          NaN         NaN         NaN
top               NaN         NaN          NaN         NaN         NaN
freq              NaN         NaN          NaN         NaN         NaN
mean        26.670190    3.509514    83.097252    1.448203    1.000000
std         13.596995    1.432231   395.983269    0.497836   11.438857
min          0.000000    1.000000     0.000000    1.000000  -99.000000
25%         17.000000    2.000000     0.000000    1.000000    1.000000
50%         29.000000    4.000000     0.000000    1.000000    2.000000
75%         38.000000    4.000000     0.000000    2.000000    2.000000
max         72.000000    7.000000  2018.000000    2.000000    6.000000

[11 rows x 21 columns]
```

Some values of `mu` are way out of range (min = -294). This is clearly from divergences in the model. We mark observations greater than 10 (in absolute value) as `nan` and then drop `nan`.

[4]:
```
# Clean estimates for mu
df['Mu'] = df['Mu'].apply(lambda x: np.nan if x < -100 else x)
df['Mu'] = df['Mu'].apply(lambda x: np.nan if x > 100 else x)
df.dropna(inplace = True)
```

1.3 Group some nationalities in others group

We keep only classes with more than 50 observations. The rest of the classes will be considered as one called “others”

[5]:
```
df["Subject_origin"].value_counts()
```

[5]:
```
2    154
6     77
5     73
9     67
8     66
0     14
7     10
1      7
3      3
4      1
Name: Subject_origin, dtype: int64
```

[6]:
```
def separate(x,y):
    if x == 2 and y == 0:
        return 11
    elif x == 2 and y == 1:
        return 12
```

```
    else:
        return x
```

[7]:
```
# There are few data on several Origins
count_origins = pd.get_dummies(df['Subject_origin']).sum()
t = 50 # threshold
df['Subject_origin'] = df['Subject_origin'].apply(lambda x: 10 if␣
↪(count_origins[x] < t) else x)
df["Subject_origin"] = df.apply(lambda x:␣
↪separate(x["Subject_origin"],x["Subject_residence"]),axis=1)
#pd.get_dummies(df['Subject_origin']).sum()
```

[8]:
```
df["Subject_origin"].value_counts()
```

[8]:
```
11    93
6     77
5     73
9     67
8     66
12    61
10    35
Name: Subject_origin, dtype: int64
```

[9]:
```
### This is just to translate the encoding to the first five integers
dicc_traslation = {10:0,11:1,12:2,5:3,6:4,8:5,9:6}
dicc_nations = {0:"Other",1:"Dominican_USA",2:"Dominican_SPA",3:"PuertoRican",4:
↪"Argentinean",5:"Moroccan",6:"Senegambian"}
#dicc_final = {0:"Other",1:"Dominican",2:"PuertoRican",3:"Argentinean",4:
↪"Moroccan",5:"Senegambian"}
df['Subject_origin'] = df['Subject_origin'].map(dicc_traslation)
```

1.3.1 Define predictors for all the inference and prediction methods

[10]:
```
predictors =␣
↪['Closeness','Clustering','Average_degree','Assortativity','Betweenness',
                ␣
↪'Closeness_origin','Closeness_residence','Number_origin','Number_residence','Mu']
target = "Subject_origin"
```

1.3.2 Define train and test split for the dataset

[59]:
```
X = df[predictors]       # independent variables
y = df[target]

test_size = 0.20 #maybe more is needed (20% is standard though)
```

```
X_train, X_test, y_train, y_test = train_test_split(X, y, test_size =␣
 ↪test_size, random_state = 0)


# Standard Scaler
sc = StandardScaler()
X_train = sc.fit_transform(X_train)
X_test = sc.transform (X_test)

#Define dataframe as merge of X and y
df_str = df[target].to_frame().merge(pd.DataFrame(sc.
 ↪fit_transform(X),columns=predictors,index=df.index), left_index=True,␣
 ↪right_index=True)
```

[12]:
```
from sklearn.linear_model import LogisticRegression
from sklearn.metrics import classification_report
log_test = LogisticRegression().fit(X_train,y_train)
predictions = log_test.predict(X_test)
print(classification_report(predictions,y_test))
```

```
              precision    recall  f1-score   support

           0       0.17      0.25      0.20         4
           1       0.40      0.38      0.39        16
           2       0.40      0.50      0.44         8
           3       0.44      0.64      0.52        11
           4       0.69      0.37      0.48        30
           5       0.24      0.40      0.30        10
           6       0.47      0.44      0.45        16

    accuracy                           0.42        95
   macro avg       0.40      0.42      0.40        95
weighted avg       0.48      0.42      0.43        95
```

2 INFERENCE

At this point, we begin to include tools of inference, beginning by the multinomial logistic regression (MLN). The library used for this analysis is mainly *statsmodels* and the main function can be checked in this link: https://stats.idre.ucla.edu/stata/dae/multinomiallogistic-regression/

In this part of the notebook we will prepare the variables, execute the regression and save the results.

2.0.1 Fit Multinomial Logistic Model

https://www.statsmodels.org/stable/generated/statsmodels.discrete.discrete_model.MNLogit.html

```
[13]: ### Uses the list 'predictors' as independent variables
formula_predictors = ' + '.join(predictors)
target_str = target +" ~ {}"
model = MNLogit.from_formula(target_str.format(formula_predictors), df_str)
results = model.fit(maxiter=200)
```

```
Optimization terminated successfully.
         Current function value: 1.387693
         Iterations 8
```

Results

```
[14]: print(results.summary())
```

```
                          MNLogit Regression Results
==============================================================================
Dep. Variable:         Subject_origin   No. Observations:                  472
Model:                        MNLogit   Df Residuals:                      406
Method:                           MLE   Df Model:                           60
Date:                Thu, 09 Jun 2022   Pseudo R-squ.:                  0.2750
Time:                        16:00:06   Log-Likelihood:                -654.99
converged:                       True   LL-Null:                       -903.45
Covariance Type:            nonrobust   LLR p-value:                 4.611e-70
================================================================================
=======
   Subject_origin=1       coef    std err          z      P>|z|      [0.025
0.975]
--------------------------------------------------------------------------------
-------
Intercept                 1.1930      0.282      4.228      0.000       0.640
1.746
Closeness                 3.2519      1.437      2.263      0.024       0.436
6.068
Clustering               -0.7151      0.255     -2.803      0.005      -1.215
-0.215
Average_degree           -3.2796      1.461     -2.245      0.025      -6.143
-0.416
Assortativity             0.8819      0.341      2.583      0.010       0.213
1.551
Betweenness              -0.6278      0.339     -1.851      0.064      -1.293
0.037
Closeness_origin          1.0520      0.328      3.205      0.001       0.409
1.695
Closeness_residence      -0.1068      0.289     -0.370      0.711      -0.672
0.459
Number_origin             1.1781      0.396      2.977      0.003       0.402
1.954
Number_residence          1.1590      0.352      3.290      0.001       0.469
```

```
1.849
Mu                     -0.4128      0.383     -1.079      0.281      -1.163
0.337
--------------------------------------------------------------------------------
-------
   Subject_origin=2       coef    std err          z      P>|z|      [0.025
0.975]
--------------------------------------------------------------------------------
-------
Intercept               0.1375      0.378      0.363      0.716      -0.604
0.879
Closeness               3.8773      1.422      2.726      0.006       1.090
6.665
Clustering             -0.1001      0.305     -0.328      0.743      -0.698
0.498
Average_degree         -5.4447      1.483     -3.672      0.000      -8.351
-2.538
Assortativity           0.8735      0.347      2.519      0.012       0.194
1.553
Betweenness            -1.0373      0.386     -2.691      0.007      -1.793
-0.282
Closeness_origin        1.2618      0.489      2.578      0.010       0.303
2.221
Closeness_residence     0.0110      0.293      0.038      0.970      -0.564
0.586
Number_origin           2.0135      0.532      3.782      0.000       0.970
3.057
Number_residence        0.8809      0.530      1.664      0.096      -0.157
1.919
Mu                     -0.6983      0.443     -1.576      0.115      -1.567
0.170
--------------------------------------------------------------------------------
-------
   Subject_origin=3       coef    std err          z      P>|z|      [0.025
0.975]
--------------------------------------------------------------------------------
-------
Intercept               0.5194      0.320      1.624      0.104      -0.107
1.146
Closeness               3.0156      1.625      1.856      0.063      -0.169
6.201
Clustering             -0.5628      0.260     -2.162      0.031      -1.073
-0.053
Average_degree         -3.1195      1.623     -1.923      0.055      -6.300
0.061
Assortativity           0.8087      0.368      2.198      0.028       0.088
1.530
Betweenness            -0.6337      0.378     -1.677      0.094      -1.374
```

```
0.107
Closeness_origin        0.6936      0.267      2.599      0.009       0.171
1.217
Closeness_residence    -0.4820      0.298     -1.615      0.106      -1.067
0.103
Number_origin          -0.3525      0.356     -0.991      0.322      -1.050
0.345
Number_residence        0.6527      0.303      2.152      0.031       0.058
1.247
Mu                      0.1057      0.366      0.289      0.772      -0.611
0.822
--------------------------------------------------------------------------------
-------
   Subject_origin=4       coef    std err          z      P>|z|      [0.025
0.975]
--------------------------------------------------------------------------------
-------
Intercept              -0.4185      0.463     -0.904      0.366      -1.326
0.489
Closeness               3.4199      1.265      2.703      0.007       0.940
5.899
Clustering              1.1115      0.340      3.269      0.001       0.445
1.778
Average_degree         -6.4449      1.415     -4.556      0.000      -9.218
-3.672
Assortativity           0.4694      0.329      1.427      0.153      -0.175
1.114
Betweenness            -0.7028      0.355     -1.978      0.048      -1.399
-0.006
Closeness_origin        0.2008      0.358      0.561      0.575      -0.501
0.903
Closeness_residence     0.5125      0.349      1.467      0.142      -0.172
1.197
Number_origin           1.1711      0.488      2.400      0.016       0.215
2.127
Number_residence        1.4349      0.426      3.371      0.001       0.601
2.269
Mu                     -0.4967      0.411     -1.210      0.226      -1.302
0.308
--------------------------------------------------------------------------------
-------
   Subject_origin=5       coef    std err          z      P>|z|      [0.025
0.975]
--------------------------------------------------------------------------------
-------
Intercept               0.5980      0.324      1.845      0.065      -0.037
1.233
Closeness               2.5844      1.328      1.945      0.052      -0.019
```

```
5.188
Clustering              0.3125      0.294      1.062      0.288      -0.264
0.889
Average_degree         -4.6305      1.402     -3.302      0.001      -7.379
-1.882
Assortativity           0.7923      0.334      2.370      0.018       0.137
1.447
Betweenness            -1.4770      0.410     -3.600      0.000      -2.281
-0.673
Closeness_origin        1.0462      0.420      2.494      0.013       0.224
1.868
Closeness_residence     0.2658      0.320      0.829      0.407      -0.362
0.894
Number_origin           1.6725      0.481      3.477      0.001       0.730
2.615
Number_residence        2.0081      0.412      4.872      0.000       1.200
2.816
Mu                     -0.4126      0.441     -0.936      0.349      -1.276
0.451
--------------------------------------------------------------------------------
-------
   Subject_origin=6       coef    std err          z      P>|z|      [0.025
0.975]
--------------------------------------------------------------------------------
-------
Intercept               0.5945      0.331      1.794      0.073      -0.055
1.244
Closeness               1.8988      1.351      1.406      0.160      -0.749
4.546
Clustering              0.1867      0.299      0.625      0.532      -0.398
0.772
Average_degree         -3.0951      1.405     -2.203      0.028      -5.849
-0.342
Assortativity           0.4756      0.334      1.423      0.155      -0.180
1.131
Betweenness            -0.9152      0.406     -2.256      0.024      -1.710
-0.120
Closeness_origin        0.1168      0.437      0.267      0.789      -0.740
0.974
Closeness_residence    -0.0413      0.289     -0.143      0.887      -0.609
0.526
Number_origin           1.6100      0.496      3.247      0.001       0.638
2.582
Number_residence        0.6063      0.475      1.277      0.202      -0.324
1.537
Mu                     -0.1930      0.434     -0.444      0.657      -1.044
0.658
================================================================================
```

=======

[15]:
```
print('pseudo r-squared = {}'.format(np.round(results.prsquared,2)))
```

```
pseudo r-squared = 0.28
```

[16]:
```
results.llr_pvalue
```

[16]:
```
4.6109000051744405e-70
```

3 PREDICTION

We train and fit a powerful non-linear (and non-parametric) machine learnin classifier to the data; a Random Forest. There are many other alternatives, but tree based metods are very powerfull and there are new techniques to help identify relevant predictors.

In this section, we want to test wether this model can outperform significantly other null (dummy) classifiers. If that is the case (which it is), it confirms the hypothesis that the predictors have relevant information about the nationalities of the subjects.

3.0.1 Train and test with MNL regression

[17]:
```
formula_predictors = ' + '.join(predictors)
model = MNLogit.from_formula(target_str.format(formula_predictors), df_str.
  ↪loc[y_train.index])
results_prediction = model.fit(maxiter=200)
ypred = results_prediction.predict(df_str.loc[y_test.index])
y_pred =list(map(np.argmax,np.array(ypred)))
##Meter función accuracy
```

```
Optimization terminated successfully.
         Current function value: 1.356241
         Iterations 8
```

[18]:
```
from sklearn.metrics import accuracy_score
print(accuracy_score(y_test, y_pred))
```

```
0.4105263157894737
```

3.0.2 Train and tune the model using k-cross fold validation

[19]:
```
scoring = 'accuracy' #'f1_macro' # This chooses the metric to optimise during␣
  ↪training (there are others!)
njobs=-1                          # This the number of cores used in your cpu␣
  ↪(-1 means "all of them")
cv=5                              # the k in k-cross-fold validation
# RANDOM FOREST
print('\nFitting Random Forest\n')
```

```
rfc=RandomForestClassifier(random_state=0)
# Parameter combinations to explore
param_grid = {
    'n_estimators': [75, 100,300,1000],
    'max_features': ['auto', None],
    'min_samples_split' :[2,6, 10, 14],
    'max_depth' : [10, 15, 30, 50,None],
    'max_samples' : [0.5 ,0.7, None],}


CV_rfc = GridSearchCV(estimator=rfc,
                  param_grid=param_grid,
                  scoring = scoring,
                  verbose=0,
                  n_jobs=njobs,
                  cv= cv)
CV_rfc.fit(X_train, y_train)

print('\nRandom Forest:')
print('Best Score: ', CV_rfc.best_score_)
print('Best Params: ', CV_rfc.best_params_)
```

```
Fitting Random Forest


Random Forest:
Best Score:  0.4668070175438597
Best Params:  {'max_depth': 10, 'max_features': 'auto', 'max_samples': 0.5,
'min_samples_split': 14, 'n_estimators': 100}
```

3.0.3 Evaluating the algorithm performance in the test set (unseen data)

[20]:
```
y_pred = CV_rfc.predict(X_test)
print('Confusion Matrix:\n ', confusion_matrix(y_test,y_pred),'\n')
print(classification_report(y_test,y_pred),'\n')
print('Accuracy: {0:.2f}'.format(accuracy_score(y_test, y_pred),2))
```

```
Confusion Matrix:
  [[ 1  1  0  0  3  1  0]
 [ 0  7  2  3  2  1  0]
 [ 0  0  3  0  2  1  4]
 [ 0  6  0  6  3  1  0]
 [ 0  0  1  0 12  2  1]
 [ 0  0  2  0  5  8  2]
 [ 0  0  4  0  5  2  4]]
```

```
              precision    recall  f1-score   support

           0       1.00      0.17      0.29         6
           1       0.50      0.47      0.48        15
           2       0.25      0.30      0.27        10
           3       0.67      0.38      0.48        16
           4       0.38      0.75      0.50        16
           5       0.50      0.47      0.48        17
           6       0.36      0.27      0.31        15

    accuracy                           0.43        95
   macro avg       0.52      0.40      0.40        95
weighted avg       0.49      0.43      0.42        95


Accuracy: 0.43
```

3.0.4 Compare this performance with null models

[21]:
```python
#  relative prevalence of each class
rel_prev = (y.value_counts() / len(y))
print(rel_prev)
```

```
1    0.197034
4    0.163136
3    0.154661
6    0.141949
5    0.139831
2    0.129237
0    0.074153
Name: Subject_origin, dtype: float64
```

[22]:
```python
# Uniform Dummy Classifier (classifies randomly with p = 1/7)

# If the classifier randomly guesses:
print('Acurracy of uniform dummy classifier: ',(((1/7) * y.value_counts()) /␣
 ↪len(y)).sum()) # = 1/6
```

```
Acurracy of uniform dummy classifier:  0.14285714285714285
```

[23]:
```python
# Stratified Dummy Classifier (classifies randomly with p ~ prevalence of each␣
 ↪class)
print('Acurracy of stratified dummy classifier: ',(rel_prev * y.value_counts()).
 ↪sum() / len(y))
```

```
Acurracy of stratified dummy classifier:  0.15125861821315714
```

[24]:
```
# Most frequent Dummy Classifier (classifies always in the most frequent class)
print('Acurracy of Most freq dummy classifier: ',rel_prev.max() )
```

```
Acurracy of Most freq dummy classifier:  0.19703389830508475
```

[25]:
```
# SKLEARN versions of the dummy classifiers (to double check and for␣
↪convinience methods)

dummy = "stratified"# most_frequent, stratified, uniform
dummy_clf = DummyClassifier(strategy=dummy,random_state=0)



# Actual accuracy of the dummy in the same train-test split as the RF model
dummy_clf.fit(X_train, y_train)
dummy_score = dummy_clf.score(X_test, y_test)
print('Mean accuracy of null ' + dummy +' model: {0:.2f}'.
↪format(dummy_score),'\n')
print('Mean accuracy (in test) of RF model: {0:.2f}'.format(CV_rfc.
↪score(X_test, y_test)),'\n')
```

```
Mean accuracy of null stratified model: 0.14

Mean accuracy (in test) of RF model: 0.43
```

[26]:
```
# Confusion matrix and report of the selected dummy classifier

y_pred_dummy = dummy_clf.predict(X_test)
print('Confusion Matrix:\n\n ',confusion_matrix(y_test,y_pred_dummy),'\n')
print(classification_report(y_test,y_pred_dummy),'\n')
print('Accuracy: {0:.2f}'.format(accuracy_score(y_test, y_pred_dummy),2))
```

```
Confusion Matrix:

  [[0 3 1 0 1 1 0]
 [1 6 1 3 0 2 2]
 [0 1 1 0 4 1 3]
 [1 3 2 1 1 5 3]
 [2 3 1 4 2 1 3]
 [2 3 6 1 1 2 2]
 [3 3 0 3 3 2 1]]

              precision    recall  f1-score   support

           0       0.00      0.00      0.00         6
           1       0.27      0.40      0.32        15
           2       0.08      0.10      0.09        10
```

```
           3       0.08      0.06      0.07        16
           4       0.17      0.12      0.14        16
           5       0.14      0.12      0.13        17
           6       0.07      0.07      0.07        15

    accuracy                           0.14        95
   macro avg       0.12      0.12      0.12        95
weighted avg       0.13      0.14      0.13        95


Accuracy: 0.14
```

[27]:

```
# Just for reference, the results of the RF Model

y_pred = CV_rfc.predict(X_test)
print('Confusion Matrix:\n\n ', confusion_matrix(y_test,y_pred),'\n')
print(classification_report(y_test,y_pred),'\n')
print('Accuracy: {0:.2f}'.format(accuracy_score(y_test, y_pred),2))
```

```
Confusion Matrix:

  [[ 1  1  0  0  3  1  0]
 [ 0  7  2  3  2  1  0]
 [ 0  0  3  0  2  1  4]
 [ 0  6  0  6  3  1  0]
 [ 0  0  1  0 12  2  1]
 [ 0  0  2  0  5  8  2]
 [ 0  0  4  0  5  2  4]]

              precision    recall  f1-score   support

           0       1.00      0.17      0.29         6
           1       0.50      0.47      0.48        15
           2       0.25      0.30      0.27        10
           3       0.67      0.38      0.48        16
           4       0.38      0.75      0.50        16
           5       0.50      0.47      0.48        17
           6       0.36      0.27      0.31        15

    accuracy                           0.43        95
   macro avg       0.52      0.40      0.40        95
weighted avg       0.49      0.43      0.42        95


Accuracy: 0.43
```

[28]:
```
dummy_report = pd.DataFrame(classification_report(y_test,dummy_clf.
↪predict(X_test), output_dict= True))

rfc_report = pd.DataFrame(classification_report(y_test,CV_rfc.predict(X_test),␣
↪output_dict= True))
```

Increase in prediction power (percentage with respect to null model) i.e. 100% means twice as good

[29]:
```
final_table = ((rfc_report - dummy_report)*100 / dummy_report).drop('support').
↪round(decimals=2)
final_table
```

[29]:
```
             0      1      2      3      4       5       6  accuracy  \
precision  inf  83.33  200.0  700.0  125.0  250.00  409.09    215.38
recall     inf  16.67  200.0  500.0  500.0  300.00  300.00    215.38
f1-score   inf  48.85  200.0  572.0  250.0  275.76  346.15    215.38

           macro avg  weighted avg
precision     345.58        275.24
recall        220.66        215.38
f1-score      240.02        223.56
```

This significant increases further support the claim that the predictors (based on ego-network properties) have useful information to predict the countries of origin of the individuals)

3.1 Shap Values

Shap values are a tool to interpret our random forest model, in this case. They tell us some intuition about which part of the prediction belongs to each feature.

A positive (negative) SHAP value indicates that the value (in this case, probability of belonging to a certain country) is reinforced (diminished) by the feature.

We will use 2 kind of plots at this moment. The first one one is a summary plot, a violin plot of the distribution of SHAP values. The colour indicates the value of the feature indicated at the left. This plot let us see the which features contribute the most (this is, they have high SHAP values). Features are ordered according to their contribution to the global prediction.

The second kind of plot you will see several times after the summary plot is the dependence plot. They show the distribution of the SHAP values of a variable. The colormap plots another variable, the one the algorithm thinks it has more interaction with the current variable. It lets us distinguish between different regimes of the coloured variable.

[30]:
```
# explain the model's predictions using SHAP
##Shap values
import shap

shap.initjs()
```

```
model = CV_rfc.best_estimator_
explainer = shap.TreeExplainer(model,X_train,check_additivity=False)
shap_values = explainer.shap_values(X_train,check_additivity=False)
```

<IPython.core.display.HTML object>

```
98%|===================| 2597/2639 [00:12<00:00]
```

3.2 Example of summary plot

We extract the summary plots that summarizes the correlations for each nationality.

SHAP values for the dominicans living in the USA

[31]:
```
dicc_nations
```

[31]:
```
{0: 'Other',
 1: 'Dominican_USA',
 2: 'Dominican_SPA',
 3: 'PuertoRican',
 4: 'Argentinean',
 5: 'Moroccan',
 6: 'Senegambian'}
```

[32]:
```
shap.summary_plot(shap_values[1],X_train,feature_names = predictors)
```

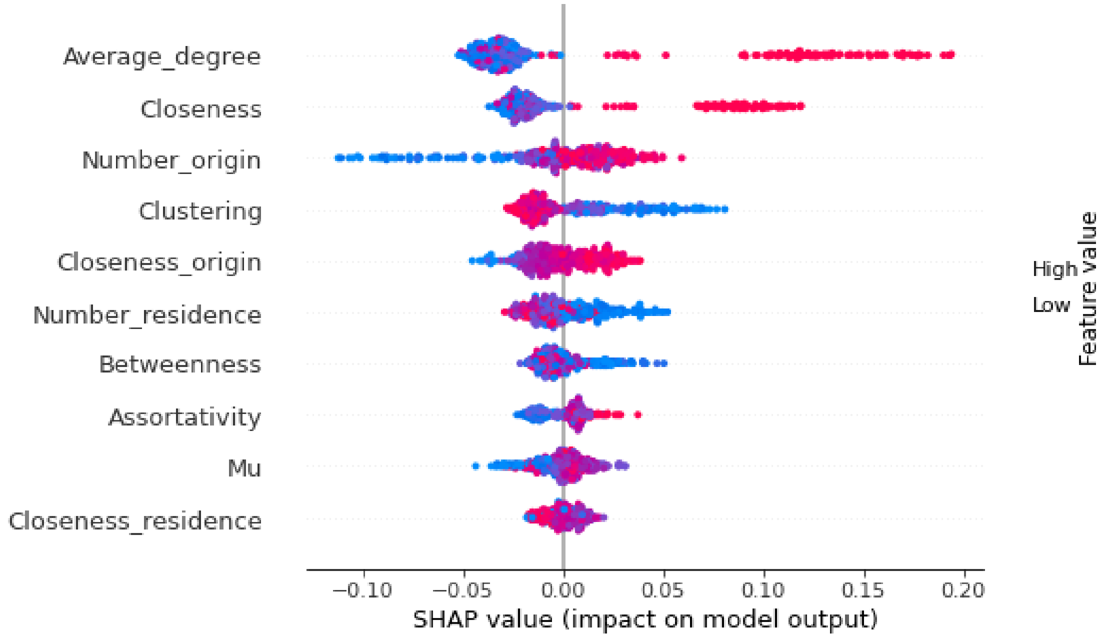

SHAP values for the dominicans living in Spain

```
[33]: shap.summary_plot(shap_values[2],X_train,feature_names = predictors)
```

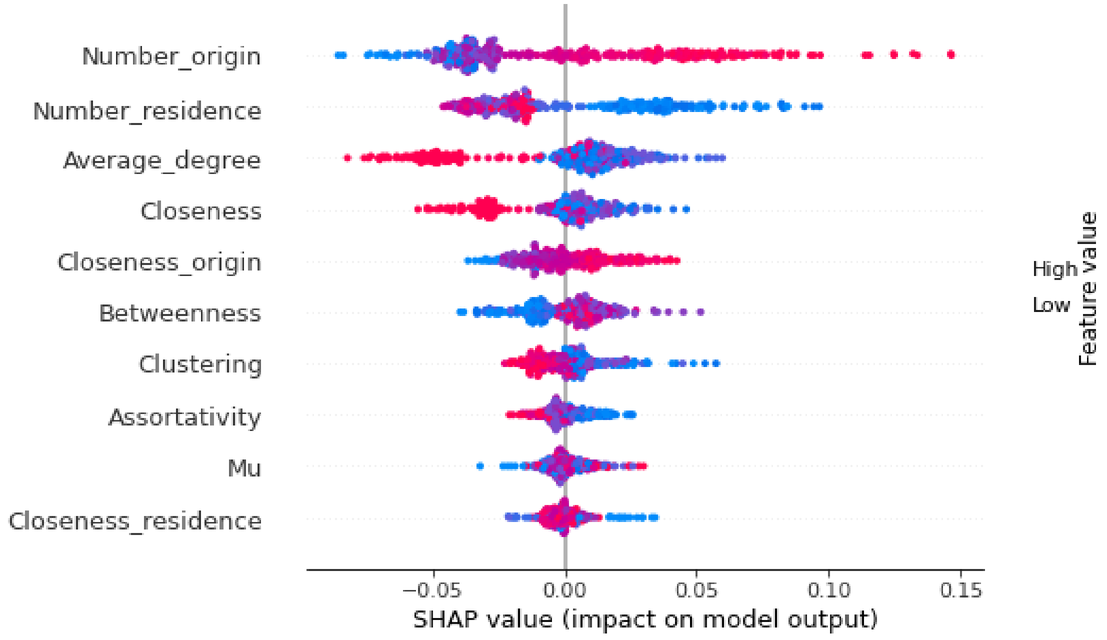

SHAP values for the Puerto Rican

```
[34]: shap.summary_plot(shap_values[3],X_train,feature_names = predictors)
```

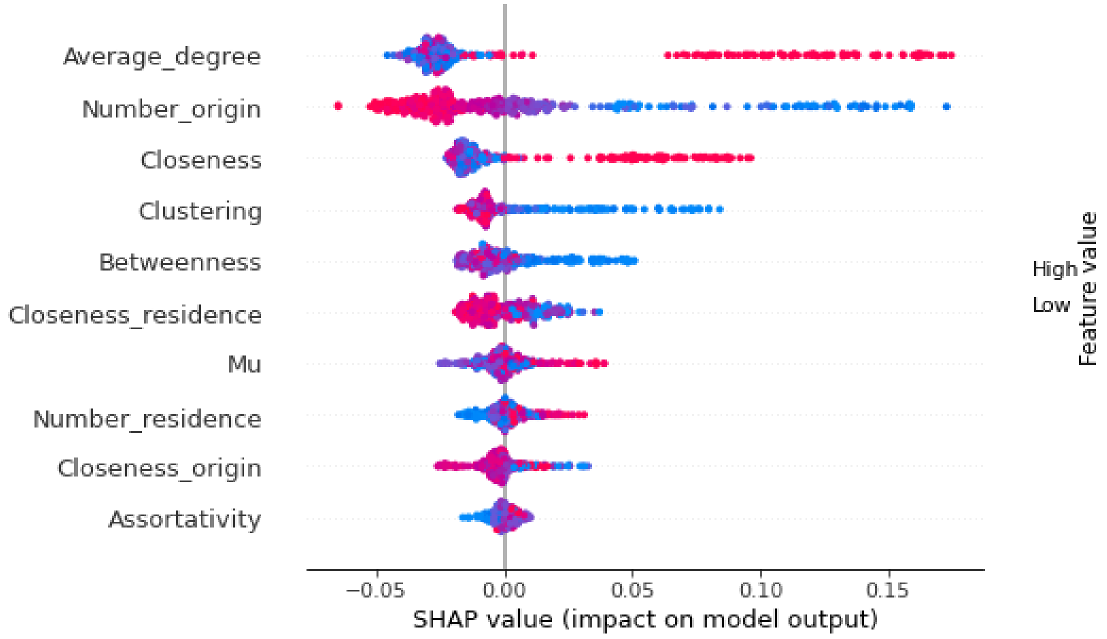

SHAP values for the argentinean

[35]:
```
shap.summary_plot(shap_values[4],X_train,feature_names = predictors)
```

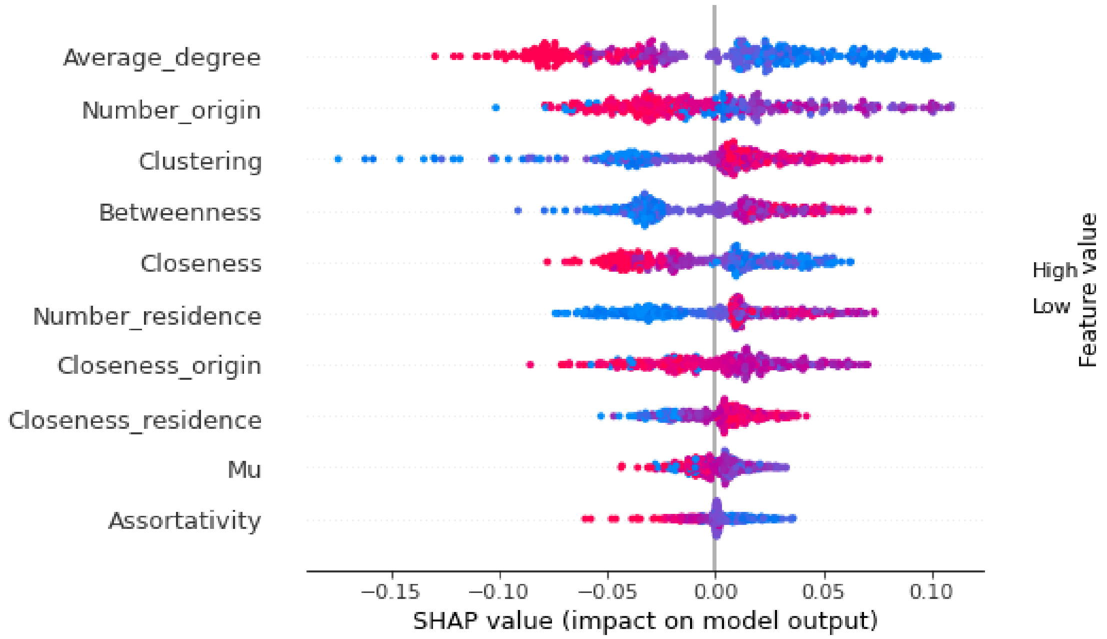

SHAP values for the moroccan

[36]:
```
shap.summary_plot(shap_values[5],X_train,feature_names = predictors)
```

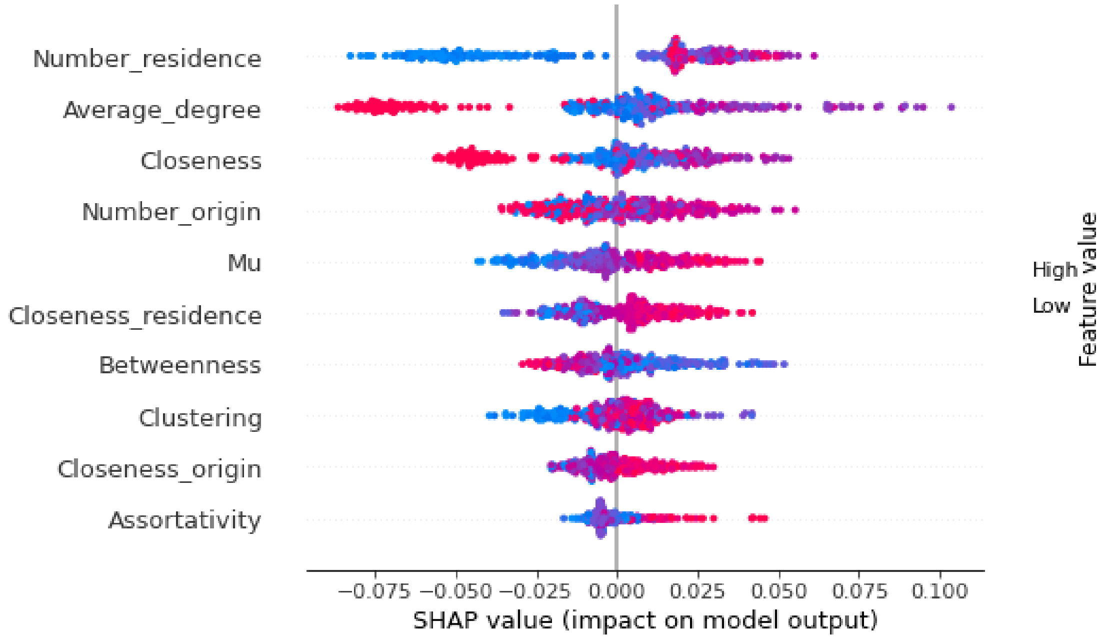

SHAP values for the senegambians

[37]:
```
shap.summary_plot(shap_values[6],X_train,feature_names = predictors)
```

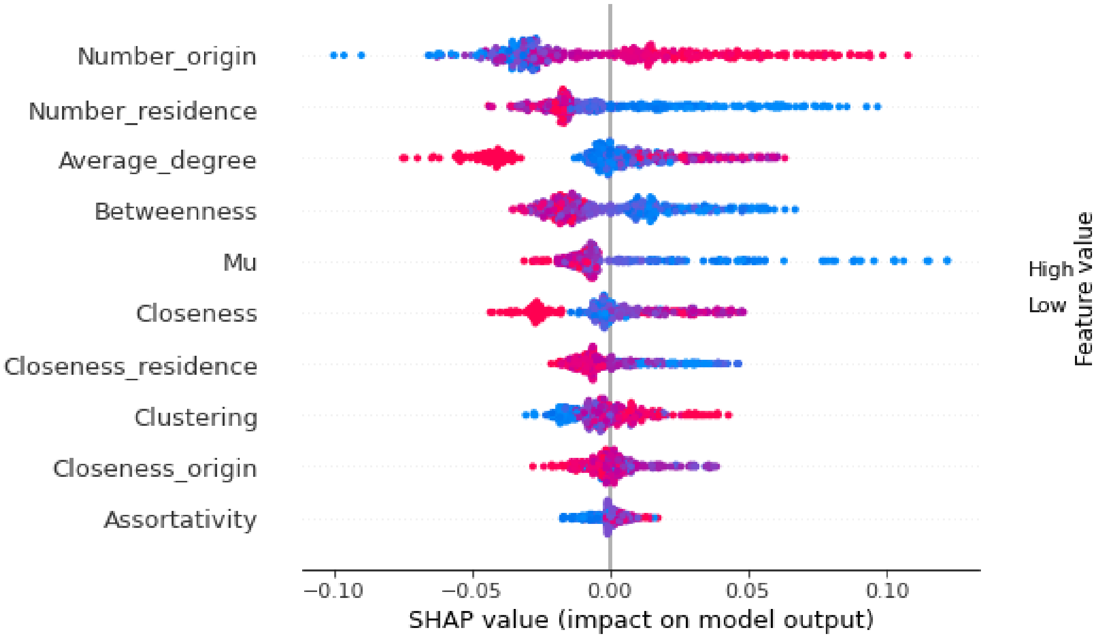

SHAP values for the control group

[38]:
```
shap.summary_plot(shap_values[0],X_train,feature_names = predictors)
```

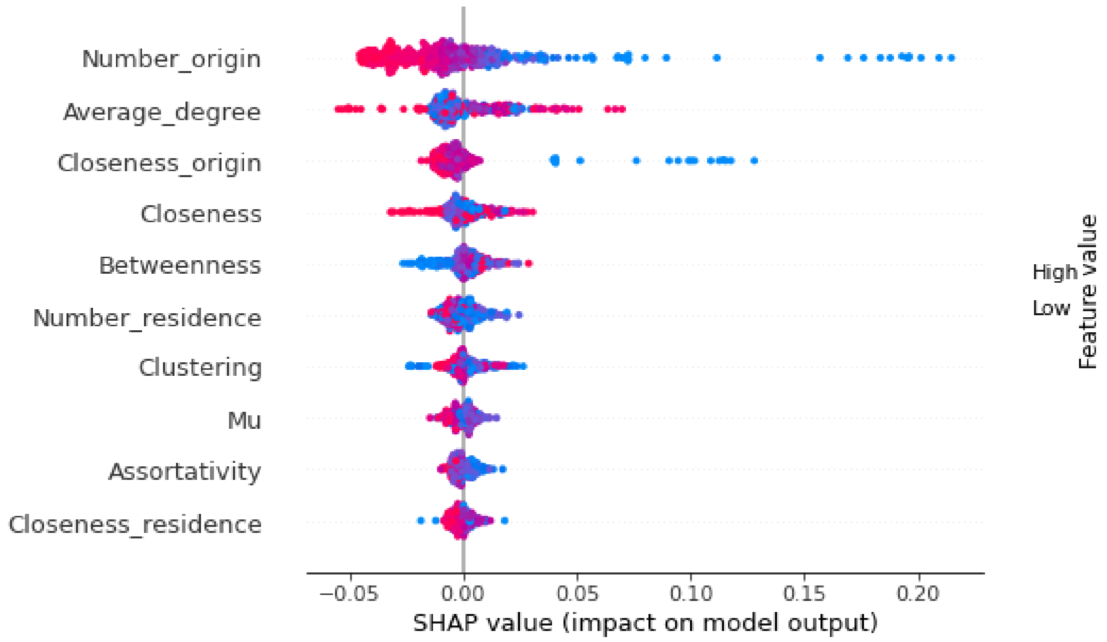

4 LIME

LIME (Local Interpretable Model-agnostic Explanations), is an algorithm that takes the decision function from the classifier (decision = f(features)). This function may be complex, but the algorithm makes a linear regression around a single prediction, weighting the importance of the coefficients with the distance to this local prediction.

This kind of algorithm helps us to explain single predictions.

[39]:
```
##Using LIME to interpret
import lime
import lime.lime_tabular
```

[40]:
```
explainer = lime.lime_tabular.LimeTabularExplainer(X_train,␣
↪feature_names=predictors, discretize_continuous=True)
```

[41]:
```
i = np.random.randint(0, X_test.shape[0])
exp = explainer.explain_instance(X_test[i], CV_rfc.predict_proba,␣
↪num_features=3, top_labels=1)
```

[42]:
```
exp.show_in_notebook(show_table=True, show_all=True)
```

```
<IPython.core.display.HTML object>
```

4.1 Artificial neural network

As a complementary method, we train a simple ANN to provide a new method and give more strength to the previous results. In order to do that, we will preprocess the data, distinguishing the categorical and numerical predictors. Then we will split the dataset into the train and test parts and, finally, we will define the model and fit to obtain a final result for the accuracy.

[43]:
```python
### Import the package tensorflow
import os
os.environ['TF_CPP_MIN_LOG_LEVEL'] = '3'
import tensorflow as tf
import logging
logging.getLogger("tensorflow").setLevel(logging.ERROR)

tf.random.set_seed(0)
```

[44]:
```python
###Define  a simple a ANN and fit our data
stat_accul = []
model_accul = tf.keras.Sequential([
    tf.keras.layers.Dense(70,activation="relu"),
    tf.keras.layers.Dense(70,activation="relu"),
    tf.keras.layers.Dense(7,activation="softmax")
])




###Compile the model
model_accul.compile(loss=tf.keras.losses.SparseCategoricalCrossentropy(),
               optimizer=tf.keras.optimizers.Adam(learning_rate=10e-4),
               metrics=["accuracy"])

### We fit the model 100 times and take notes of the accuracy on the test set

history_accul = model_accul.fit(X_train,
                         np.array(y_train),
                         epochs=100,
                         verbose = 0)
stat_accul.append(model_accul.evaluate(X_test,np.array(y_test))[1])
```

```
3/3 [==============================] - 0s 1ms/step - loss: 1.9304 - accuracy:
0.4526
```

4.2 Display the final results

[45]:
```python
print(f"The final results for a training iteration is {np.average(stat_accul):.
 ↪2f}")
```

```
The final results for a training iteration is 0.45
```

4.3 Radar plots for regressions

[46]:
```
from sklearn.preprocessing import MinMaxScaler
sc = MinMaxScaler()
df_fitted = sc.fit_transform(results.params)

df_polar = pd.DataFrame(sc.fit_transform(results.params.transpose())).
 ↪transpose()
df_polar.columns = list(dicc_nations.values())[1:]
#df_polar.index = predictors.insert(0,"Intercept")
df_polar = df_polar.drop(0,axis=0).reset_index().drop("index",axis = 1)
df_polar.index = predictors
```

[47]:
```
import plotly.graph_objects as go
import plotly.io as pio
pio.renderers.default = "notebook+pdf"

categories = predictors


fig = go.Figure()
for col in df_polar.columns[:2] :

    fig.add_trace(go.Scatterpolar(
        r = df_polar[col].values,
        theta = categories,
        fill = "toself",
        name = col
    ))



fig.update_layout(
  polar=dict(
    radialaxis=dict(
      visible=True,
      range=[0, 1]
    )),
  showlegend=True
)

fig.show()
```

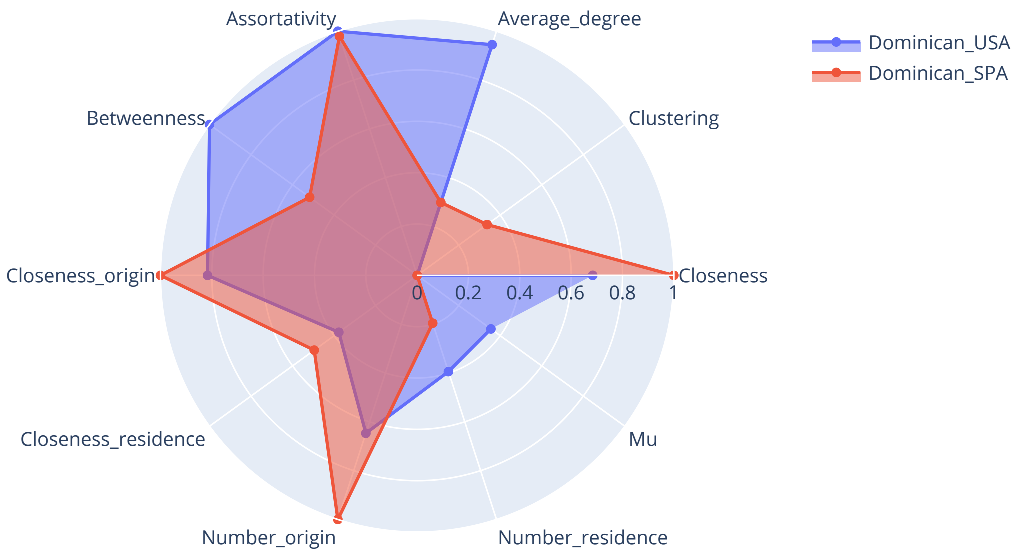

Loading [MathJax]/extensions/MathMenu.js

Tomando el trozo de dataset que corresponde a los dominicanos, vamos a ver dónde los colocan y con quien los confunde cada uno de los métodos.

[64]:
```
X_prueba = df[(df["Subject_origin"]==1) | (df["Subject_origin"] ==␣
↪2)][predictors].values     # independent variables
y_prueba = df[(df["Subject_origin"]==1) | (df["Subject_origin"] == 2)][target].
↪values

X_prueba = sc.transform(X_prueba)
```

Random Forest

[95]:
```
test_1 = confusion_matrix(y_prueba,CV_rfc.predict(X_prueba))
for i,row in enumerate(test_1[:2]):
    most_probable = dicc_nations[np.where(row ==␣
↪sorted(row,reverse=True)[1])[0][0]+1]
    print(f"A {dicc_nations[i+1]} person is probably missmatched with a␣
↪{most_probable} person")
```

```
A Dominican_USA person is probably missmatched with a PuertoRican person
A Dominican_SPA person is probably missmatched with a Senegambian person
```

Multinomial Logistic regression

```
[101]: test_1 = confusion_matrix(y_prueba,model.predict(X_prueba))
for i,row in enumerate(test_1[:2]):
    most_probable = dicc_nations[np.where(row ==␣
 ↪sorted(row,reverse=True)[1])[0][0]+1]
    print(f"A {dicc_nations[i+1]} person is probably missmatched with a␣
 ↪{most_probable} person")
```

```
A Dominican_USA person is probably missmatched with a PuertoRican person
A Dominican_SPA person is probably missmatched with a Senegambian person
```

Neural network

```
[106]: test_1 = confusion_matrix(y_prueba,model_accul.predict(X_prueba).argmax(axis=1))
for i,row in enumerate(test_1[:2]):
    most_probable = dicc_nations[np.where(row ==␣
 ↪sorted(row,reverse=True)[1])[0][0]+1]
    print(f"A {dicc_nations[i+1]} person is probably missmatched with a␣
 ↪{most_probable} person")
```

```
A Dominican_USA person is probably missmatched with a PuertoRican person
A Dominican_SPA person is probably missmatched with a Senegambian person
```

```
[ ]:
```
